# Contrasting effects of copper limitation on the photosynthetic apparatus in two strains of the open ocean diatom *Thalassiosira oceanica*

**DOI:** 10.1371/journal.pone.0181753

**Published:** 2017-08-24

**Authors:** Anna A. Hippmann, Nina Schuback, Kyung-Mee Moon, John P. McCrow, Andrew E. Allen, Leonard J. Foster, Beverley R. Green, Maria T. Maldonado

**Affiliations:** 1 Department of Earth, Ocean, and Atmospheric Sciences, University of British Columbia, Vancouver, British Columbia, Canada; 2 Department of Biochemistry & Molecular Biology, University of British Columbia, Vancouver, British Columbia, Canada; 3 Department of Microbial & Environmental Genomics, J. Craig Venter Institute, La Jolla, California, United States of America; 4 Department of Botany, University of British Columbia, Vancouver, British Columbia, Canada; Auburn University, UNITED STATES

## Abstract

There is an intricate interaction between iron (Fe) and copper (Cu) physiology in diatoms. However, strategies to cope with low Cu are largely unknown. This study unveils the comprehensive restructuring of the photosynthetic apparatus in the diatom *Thalassiosira oceanica* (CCMP1003) in response to low Cu, at the physiological and proteomic level. The restructuring results in a shift from light harvesting for photochemistry—and ultimately for carbon fixation—to photoprotection, reducing carbon fixation and oxygen evolution. The observed decreases in the physiological parameters F_v_/F_m_, carbon fixation, and oxygen evolution, concomitant with increases in the antennae absorption cross section (σ_PSII_), non-photochemical quenching (NPQ) and the conversion factor (φ_e:C_/η_PSII_) are in agreement with well documented cellular responses to low Fe. However, the underlying proteomic changes due to low Cu are very different from those elicited by low Fe. Low Cu induces a significant four-fold reduction in the Cu-containing photosynthetic electron carrier plastocyanin. The decrease in plastocyanin causes a bottleneck within the photosynthetic electron transport chain (ETC), ultimately leading to substantial stoichiometric changes. Namely, 2-fold reduction in both cytochrome *b*_*6*_*f* complex (cyt*b*_*6*_*f*) and photosystem II (PSII), no change in the Fe-rich PSI and a 40- and 2-fold increase in proteins potentially involved in detoxification of reactive oxygen species (ferredoxin and ferredoxin:NADP^+^ reductase, respectively). Furthermore, we identify 48 light harvesting complex (LHC) proteins in the publicly available genome of *T*. *oceanica* and provide proteomic evidence for 33 of these. The change in the LHC composition within the antennae in response to low Cu underlines the shift from photochemistry to photoprotection in *T*. *oceanica* (CCMP1003). Interestingly, we also reveal very significant intra-specific strain differences. Another strain of *T*. *oceanica* (CCMP 1005) requires significantly higher Cu concentrations to sustain both its maximal and minimal growth rate compared to CCMP 1003. Under low Cu, CCMP 1005 decreases its growth rate, cell size, Chl*a* and total protein per cell. We argue that the reduction in protein per cell is the main strategy to decrease its cellular Cu requirement, as none of the other parameters tested are affected. Differences between the two strains, as well as differences between the well documented responses to low Fe and those presented here in response to low Cu are discussed.

## Introduction

Diatoms account for almost a quarter of global primary productivity, thus contributing significantly to the transfer of CO_2_ from the atmosphere to the ocean interior [1–3]. Yet in large oceanic regions, optimal growth of diatoms is constrained by iron (Fe) supply [4]. However, in these low Fe regions, diatoms and other phytoplankton persist, and have evolved unique physiological strategies to grow at chronically low Fe levels [5–10], even though they also respond rapidly to sporadic Fe inputs [11,12]. In phytoplankton, the process of photosynthesis has the highest requirement for Fe. Therefore, some of the underlying cellular adaptations to low Fe include a) reducing the number of chloroplasts, and the volume of individual chloroplasts [13], b) restructuring the photosynthetic apparatus, such that the Fe intensive PSI is down-regulated relative to PSII [9], and c) replacing the electron carrier ferredoxin with the non-metal equivalent flavodoxin [14]. In addition to these physiological adaptations under low Fe, open ocean diatoms enhance their demand for copper (Cu)—another essential redox active metal—by eliminating the Fe-containing cyt*c*_*6*_ in photosynthesis and replacing it with plastocyanin, a Cu-containing counterpart [15], and by up-regulating a high-affinity Fe uptake system (HAFeT) that depends on a multi-Cu oxidase [8,16]. These physiological adaptations result in a higher Cu demand in Fe-limited than in Fe-sufficient diatoms, and suggest an intricate link between Fe and Cu physiology [17].

Open ocean Fe concentrations are significantly lower (average ~0.6 nM) than those at the coast (average ~ 2nM) [18]. Diatoms in oligotrophic regions, such as the Sargasso Sea, although not primarily chronically Fe limited, often may experience low Fe availability (surface waters [Fe]_diss_ ranging from 0.2–0.8 nM [19], relative to the average half-saturation constant for growth for Fe (K_u_) for field populations, 0.32 nM Fe [20]) in addition to macronutrient limitation and might, as a result, exhibit the physiological adaptations mentioned above. Indeed, several laboratory and field studies have shown that open ocean phytoplankton usually have higher Cu:C demands and thus are more easily limited by low Cu than their coastal counterparts [16,17,21].

Literature on adaptations of phytoplankton to cope with Cu limitation is scarce. In the model freshwater green alga *Chlamydomonas reinhardtii*, Cu limitation induces a tight transcriptional regulatory mechanism [22,23]. Most notably, it uses the Cu-containing electron carrier plastocyanin when grown under Cu replete conditions and switches to the Fe-containing cyt*c*_*6*_ under Cu limiting conditions [24,25]. This decreases its cellular photosynthetic Cu demand and enables adequate Cu supply to cytochrome oxidase in respiration [26]. An increase in unsaturated fatty acids in the thylakoid membrane also occurs in response to Cu limitation [22]. Changes in thylakoid fatty acid composition can have far reaching consequences in a photosynthetic cell [27–29]. An increase in the fatty acid MGDG (monogalactosyldiacylglycerol) might result in an increase in non-photochemical quenching (NPQ, an estimate of how much excess excitation energy is dissipated as heat) by facilitating both recruitment of diadinoxanthin and its interaction with light harvesting complexes (LHCs) [28,30,31]. One study examined the effects of low Cu on the photosynthetic apparatus of the prymnesiophyte *Phaeocystis*. Photosystem II reaction centers seemed to be damaged but no effects were observed downstream in the electron transport chain [32].

In addition to photosynthesis, Cu limitation affects diatom physiology by a) inducing a high-affinity Cu uptake system, which has different characteristics and regulation in oceanic and coastal centric species [33], and b) increasing the cellular Chl*a* and lipid content in pennate diatoms [34]. Most recently, the genes encoding various Cu transporters and chaperones have been identified *in silico* in the genome of the diatom *T*. *pseudonana* [35].

Many of the physiological adaptations to Fe limitation described above were investigated using the model open ocean centric diatom, *T*. *oceanica*, strain CCMP 1003 (hereafter referred to as TO03), whose genome has not been sequenced. However, recently, the genome of a different strain of *T*. *oceanica* (CCMP 1005; here TO05) was sequenced [13]. Given the importance of Cu nutrition for *T*. *oceanica* [15–17,21,33], we aimed to investigate the physiological and proteomic response to Cu limitation in both strains. Since TO03 has not been sequenced, we grew this strain under various Cu conditions, and used transcriptomics to create an expressed sequence tag (EST) library with assembled contigs. Our preliminary growth experiments showed very contrasting Cu demands between TO05 and TO03 and inspired further comparative investigations. The present study focuses on the physiological and proteomic response of the photosynthetic apparatus to Cu limitation in both strains. Here, we also discuss similarities and differences between the well-known photosynthetic response of diatoms to low Fe and their understudied response to low Cu.

## Methods

### Study organism

Two strains of the centric diatom species *Thalassiosira oceanica* were used in this investigation, CCMP 1003 and CCMP 1005. Species and strain were confirmed based on the ITS region sequence (encompassing the ITS1–5.8S –ITS2, [36,37]; S1 Sequence). All isolates were obtained from the Provasoli-Guillard Center for Culture of Marine Phytoplankton, now National Centre for Marine Algae and Biota (NCMA) at Bigelow Laboratory for Ocean Sciences.

### ITS region

For DNA extraction, the DNeasy^®^ Plant Mini Kit (cat. nos. 69104 and 69106) from QUIAGEN was used following its manual with one modification: cells from 40 mL of late exponential culture were concentrated via centrifugation, then mixed with 400 μL AP buffer, 2 μL RNAse and 100 μL 0.2–0.5mm silica microbeads. The microbeads increase the DNA yield by breaking open the silica frustules. The 700bp long ITS fragment, comprised of ITS1, 5.8S rDNA gene and part of ITS2, as described by Moniz and Kaszmarska [36], was amplified using primers ITS1 and ITS5 and a PCR reaction mix as described by White *et al*. [38] with an amplification regime as per Amato *et al*. [39]. PCR products were visualized in a 1.5% agarose gel (S1 Fig). For PCR product clean up, Promega Wizard ^®^ SV Gel and PCR Clean-Up System (#A9282) was used. Sequencing was conducted by Genewiz (Seattle, USA), following their standard procedure. The resulting sequences were aligned with other published *Thalassiosira* ITS regions using the multiple sequence alignment algorithm MAFFT [40,41].

### Culture media and growth conditions

Cultures were grown in the artificial seawater medium Aquil [42], which consists of a salt mixture (synthetic ocean water, SOW) at pH 8.2, enriched with standard additions of nitrate (300 μmol L^-1^ NO_3_^-^), phosphate (10 μmol L^-1^ PO_4_^3-^), and silicic acid (100 μmol L^-1^ SiO_3_^2-^), as well as vitamins. The trace elements Mn, Zn, Co, Mo, and Se were added bound to EDTA to attain a final EDTA concentration of 100 μmol L^-1^ in the growth medium [43]. Iron and Cu were added separately as a premixed FeEDTA or CuEDTA complex (1:1.05). Iron was added at a total concentration of 1.37 μmol L^-1^ (pFe 19) (speciation calculated using MINEQL) [44]. Copper was added to give total concentrations of 10.2 nmol L^-1^ (pCu 14) and 14.32 nmol L^-1^ (pCu 13.5) in the Cu-sufficient media for TO03 and TO05, respectively. Cu-limited media had just background Cu contamination for TO03 and was enriched with 6.08 nmol L^-1^ for TO05. Background Cu contamination in the media was <1 nmol L^-1^, and was determined in parallel medium preparations by chemiluminescence detection using a flow injection analysis system [45,46]. Sterile, trace metal-clean techniques were used during all manipulations, and the metal-EDTA reactions in the media were allowed to equilibrate overnight.

### Whole cell physiology

#### Growth measurements

Both strains were cultured in 28 mL acid-cleaned polycarbonate tubes at 19 ±1°C in continuous light (ca. 155 μmol quanta m^-2^ s^-1^). Growth was monitored by daily measurements of in vivo Chl*a* fluorescence using a Turner10-AU Fluorometer, and cultures were kept in exponential growth phase using semi-continuous batch culturing [47]. The cultures were considered acclimated when growth rates during approximately 40 cell divisions (five successive transfers), varied by <15% [47]. Acclimated, exponentially growing cells were used to inoculate triplicate 10 L cultures grown in polycarbonate carboys with gentle stirring. Growth in the 10 L cultures was determined by monitoring cell density with a Coulter Counter (model Z2). During early to mid-exponential phase, each biological replicate culture was sampled in duplicate for Chl*a* concentration, total cellular protein, oxygen evolution, Fe uptake, ^14^C uptake, ^14^C light response (P*vs*E, PE) curves, electron transport rate in the reaction centre of PSII (ETR_RCII_) -PE curves and other FRRF derived parameters, as described below. The remainder of the culture (7–8 L) was used for differential proteomic analyses. Sterile, trace metal clean techniques were used at all times.

#### Chl*a* measurements

Duplicate 10 mL subsamples of the 10 L cultures were filtered onto a 25 mm glass fiber filter (GF/F, pore size 3μm), flash frozen in liquid N_2_ and stored at -20°C until analysis. Chlorophyll was extracted overnight in 8 mL ice-cold 90% acetone and the Chl*a* concentration was determined fluorometrically following the method of Arar and Collins [48].

#### Cellular protein concentration

Cellular protein concentration was measured following a method adapted from Lommer *et al*. [13]. Briefly, 1.5 x 10^6^ cells were concentrated by filtering 10–20 mL of culture onto a 25 mm, 3 μm pore-sized polycarbonate (PC) filter. Filters were flash frozen in liquid N_2_ and stored at -80°C until analysis. For protein determination, cells were resuspended and lysed in 250 μL SDS/CO_3_ buffer [4% w/v SDS, 68mM Na_2_CO_3_, Halt^™^ protease inhibitor cocktail (Thermo Fisher Scientific, Waltham, MA, USA, #78430)] with application of ultrasonication. Cell debris was pelleted via centrifugation at 16,000 x g, at room temperature, for 4 min; protein concentration was determined with technical duplicates for each of the three biological replicates, using 50 μl of the supernatant in a bicinchoninic acid (BCA) protein assay (Thermo Fisher Scientific, Waltham, MA, USA, # 23227).

#### Oxygen evolution

Oxygen evolution was measured using a Clark-type oxygen electrode (Hansatech). Before each experiment, the electrode was calibrated with sterile SOW bubbled with filtered compressed air or N_2_ gas for O_2_ saturated and O_2_ zero standard, respectively. Cells were concentrated 15-fold via gentle filtration onto a 3 μm pore-sized PC filter, resuspended in fresh media, and duplicate subsamples from each biological replicate were introduced to the sample chamber. Samples were briefly bubbled with N_2_ before illumination at growth irradiance while O_2_ evolution was recorded. Subsequently, samples were exposed to complete darkness, in order to record the rate of respiration. Rates of oxygen evolution and respiration were derived from the slope of the linear increase and decrease of dissolved dioxygen (O_2_) over time. The raw data was analyzed using the software Oxypeak supplied by the manufacturer.

#### Chl*a* fluorescence parameters and ETR_RCII_ using fast repetition rate fluorometry (FRRf)

A bench-top FRRf instrument (Soliense Inc.) was used for all active chlorophyll fluorescence (ChlF) measurements as described in detail in Schuback *et al*. [49]. Briefly, a single turnover (ST) protocol was designed and used to derive the ChlF yields F_o_ and F_m_ in dark-regulated state, as well as F′ and F_m_′ in the light-regulated state while subjected to 10 ‘background’ irradiances ranging from 0 to 1,000 μmol quanta m^-2^ s^-1^. F_o_′ was calculated as F_o_′ = F_o_/ (F_v_/F_m_ + F_o_/F_m_′) [50]. The five ChlF yields, F_o_, F_m_, F′, F_m_′ and F_o_′ were used to calculate ChlF parameters following [51], as described in detail in Schuback *et al*. [49].

For the dark-regulated state, we derived the commonly used F_v_/F_m_ ratio as F_v_/F_m_ = (F_m_-F_o_) / F_m_ [52]. Furthermore, for each ‘background’ light level we derived: (1) The photochemical quenching of variable fluorescence, Fq′/Fv′ = (Fm′-F′) / (Fm′-Fo′), which quantifies the fraction of functional RCII in the open state (i.e. primary quinone acceptor Q_A_ in the oxidized state); (2) The maximum quantum yield of PSII photochemistry, Fv′/Fm′ = (Fm′-Fo′) / Fm′, which can be used to quantify the extent to which photochemistry in PSII is limited by competition with thermal decay of excitation energy [50]; (3) The overall quantum efficiency of photochemical energy conversion in PSII at a given light intensity Fq′/Fm′ = (Fm′-F′) / Fm′ = ФPSII′ (the product of Fq′/Fv′ and Fv′/Fm′). The functional absorption cross section of PSII, σ_PSII_ (Å^2^ RCII^-1^), was derived from the rate of closure of RCII in the dark-regulated and at each light-regulated state [52,53]. Rates of charge separation (ETR_RCII_) in functional RCII (mol e^-^mol RCII^-1^ s^-1^) were estimated as the product of incident irradiance (E, μmol quanta m^-2^ s^-1^), the fraction of irradiance absorbed by PSII (σ_PSII_, Å^2^ RCII^-1^) and the efficiency with which charge separation occurs in RCII (Fq′/Fv′):

$$ETR_{RCII}=E\times \sigma_{PSII}{}^{\prime }\times Fq^{\prime }/{\text{Fv}}^{\prime }\times 6.022\times 10^{-3}$$ (1)

The number 6.022 x 10^−3^ converts μmol quanta to quanta, Å^2^ to m^2^, and RC to mol RC.

Non-photochemical quenching (NPQ) at each light level was estimated as the normalized Stern-Volmer quenching coefficient, defined as NPQ_NSV_ = (F_m_′/F_v_′) -1 = F_o_′/F_v_′ [49,54,55].

#### ^14^C carbon assimilation

Light response curves (Photosynthesis *vs* irradiance E, PE curves) for C fixation were generated by measuring rates of carbon assimilation at various light intensities as described in Schuback *et al*. [49], using a custom built photosynthetron [56]. A photosynthetron is a temperature controlled apparatus in which small aliquots of the same culture are subjected to different light intensities simultaneously. Briefly, on the day of harvest, an 80 mL subsample of exponentially growing culture was spiked with 40 μCi NaH^14^CO_3_ and 3 mL aliquots were incubated in glass vials in the photosynthetron for 60 minutes. Each subsample was subjected to a different light intensity provided by high power light emitting diodes (LEDs) located under each glass vial. Each PE curve consisted of 11 light levels spanning intensities from 2 to 700 μmol quanta m^-2^ s^-1^. Actual light intensities were measured before and after each experiment using a 4π quantum sensor (QSL-2100, Biospherical Instruments) immersed in water inside a scintillation vial. Temperature was kept within 1°C of growth temperature by circulating water from a water-bath through an aluminum cooling jacket. Duplicate curves were measured for each sample. The incubation was terminated by adding 1 mL of 1 M HCl to each vial. The samples were then completely dried, and subsequently re-suspended in 1 mL MilliQ water.

#### PE curves

Measurements of CO_2_-assimilation and ETR_RCII_ were plotted against irradiance, and the model of Jassby and Platt [57] was fit to the data using the ‘phytotool’ package [58] in R and RStudio [59,60]. For both rates of productivity, we derived the light utilization efficiency α [ETR_α_ = (mol e^-^ RCII^-^) / (μmol quanta m^-2^s^-1^); ^14^C_α_ = (g C g Chl*a*^-1^ h^-1^) / (μmol quanta m^-2^ s^-1^)], the light saturated maximum rate P_max_ (ETR_Pmax_ = mol e^-^ RCII^-^_;_^14^C_Pmax_ = g C g Chl*a*^-1^ h^-1^), and the light saturation point E_k_ (μmol quanta m^-2^ s^-1^). When photoinhibition was observed at high irradiances, the data points were excluded from the fitting procedure.

#### Derivation of conversion factor

Because we derived ETR_RCII_ in units of mol e^-^ mol RCII^-1^ s^-1^ and CO_2_-assimilation in units of mol C mol Chl*a*^-1^ s^-1^, the conversion factor between the two rates accounts for changes in Chl*a* functionally associated with each RCII (1/n_PSII_, mol Chl*a* mol RCII^-1^) and the number of charge separations in RCII needed per CO_2_-assimilated into organic carbon products (Φe:C, mol e^-^ mol C^-1^).

$$\frac{{\text{ETR}}_{\text{RCII}}\left({\text{mol e}}^{-}{\text{ mol RC}}^{-1}{\text{s}}^{-1}\right)}{{\text{CO}}_{2}\text{ assimilation }\left(\text{mol C mol Chl }a^{-1}{\text{s}}^{-1}\right)}={\text{ Φ}}_{\text{e}:\text{C }}\left(\frac{{\text{mol e}}^{-}}{\text{mol C}}\right)\;\times \;\frac{1}{{\text{n}}_{\text{PSII}}}\left(\frac{\text{mol Chl }a}{\text{mol RCII}}\right)$$ (2)

In this approach, we attribute the observed decoupling between ETR_RCII_ and CO_2_-assimilation to changes in both 1/n_PSII_ and Φ_e:C_.

#### Statistical analysis of physiological experiments

The dataset presented in this paper (replete and Cu-limiting conditions, raw data in S1 Data) is a subset of a larger dataset (not published, *manuscript in prep*.), as cultures were also grown in Fe-limiting and Fe/Cu co-limiting conditions. Statistical analysis to test for main and interacting effects was done on this complete dataset to decrease the number of false positives that would ensue when examining each of the three treatment pairs separately (ctrl *vs*. low Cu, ctrl *vs*. low Fe, ctrl *vs*. low FeCu). The three main factors included in the linear model were: 1) Strain (TO03 *vs*. TO05), 2) Fe level (high *vs*. low), and 3) Cu level (high *vs*. low). We used the phia package [61] in R and RStudio [59,60] to fit the linear model (including all three main effects) as well as for the post-hoc analysis of interactions (phia) in the factorial ANOVA. To focus the analysis on the low Cu data presented here, we then tested simple main effects for interactions, which consisted in evaluating contrasts across the levels of one factor, while the values of the other interaction factors were fixed at certain levels. For example, to test if the low Cu response in TO03 is significantly different from the control treatment, we set the fixed factors “Strain” = TO03, and “Fe level” = high. The variable factor to be tested would be “Cu level” (high *vs*. low). To test for differences between strains under low Cu, the factors of variable “Strain” (contrasts of TO03 *vs*. TO05) would be evaluated with fixed “Fe level” = high and “Cu level” = low. Note, that the Fe level is always set as fixed factor and always as “high” as we are only interested in changes in the response to low Cu.

### Transcriptomics

#### RNA-seq library preparation

Total RNA was purified from cell pellets using the Trizol reagent (Life Technologies; Carlsbad, CA), treated with DNase (Qiagen, Valencia, CA, USA), and cleaned with the RNeasy MinElute Kit (Qiagen, Valencia, CA, USA). RNA-Seq libraries were constructed with TruSeq RNA Sample Preparation Kits (Illumina; San Diego, CA). 0.8 μg of total RNA was used as input followed by the manufactures TruSeq RNA Sample Preparation Low Throughput protocol. A high sensitivity DNA Assay chip was used to assess quality (Agilent; Santa Clara, CA, USA). The mean sizes of the libraries were around 430-480bp.

#### RNA-seq data analysis

Transcriptomes were sequenced on the Illumina HiSeq. Reads were trimmed of primer sequences, and rRNA sequences removed using Ribopicker v.0.4.3 [62]. Contigs were assembled *de novo* using CLC Assembly Cell (version 3. 22.55708) [63]. Putative open reading frames on the assembled contigs were called using FragGeneScan [64]. Predicted protein sequences were annotated by hidden markov models for Pfam and TIGRfam, and by Blastp against PhyloDB 1.06 with an E-value threshold of 1e^-3^. PhyloDB is a comprehensive in-house database at JCVI consisting of proteins from many public sequence databases, including KEGG [65], Pfam [66], GenBank [67], Ensembl [68]and several in-house assemblies of algal uniculture transcriptomic sequences. The database PhyloDB is available for download at the following website: https://scripps.ucsd.edu/labs/aallen/data/. The RNA-seq data reported here have been deposited in the NCBI sequenceread archive (https://www.ncbi.nlm.nih.gov/sra/; BioProject accession no. PRJNA382002; BioSample accession nos. SAMN06698917- SAMN06698931).

#### *T*. *oceanica* strain comparison

Filtered transcriptome reads from TO03 (CCMP1003) were mapped to the following sources: predicted ORFs resulting from the *de novo* assembly of TO03, assembled contigs of the publicly available TO05 genome (CCMP 1005, https://www.ncbi.nlm.nih.gov/bioproject/PRJNA36595, downloaded 12/1/2015), nuclear genes of TO05, and chloroplast genes of TO05. Read mapping was performed using BWA MEM [69] with default parameters. Mapped reads from each sample were counted and reads per kilobase of transcript per million mapped reads (RPKM) values were calculated for all genes.

### Proteomics

#### Protein purification

From the 10 L triplicate cultures, 7–8 L in early to mid-exponential growth phase were harvested via gentle filtration onto 47 mm, 3 μm pore-sized PC filters. Depending on cell density and culture treatment, filters clogged after varying amounts of culture volume and had to be replaced. Cells washed from all filters were combined and resuspended in 10 mL ice cold SOW and concentrated via centrifugation at 2,500 x g for 10 min. The pellet was resuspended in 2 mL ice cold SOW and spun at 13,000 x g for 2 min. The resulting cell pellet was flash frozen in liquid N_2_ and stored at -80°C till final processing. During protein extraction, all samples and buffers were kept on ice. To disrupt the cells, approximately 0.8 g of glass beads (212–300 μm diameter, Sigma, #G1277-100g) were added to the cell pellet in the Eppendorf tube, as well as ~ 1.5 mL lysis buffer (0.05 M Hepes, 0.02 M KCl, 0.001 M EDTA, 0.0002 M DTT, 0.15 Sorbitol plus freshly added Protease Inhibitor, one Roche^®^ Tablet per 50 mL lysis buffer). Complete cell lysis was achieved with three 1 min vortexing intervals, interrupted by 1 min cooling periods on ice. The samples were then settled by10 seconds centrifugation in a tabletop centrifuge and the supernatant was transferred to a new 2 mL Eppendorf tube. The remaining cell debris and beads were repeatedly washed with lysis buffer and the supernatants were combined until the wash steps resulted in an almost clear supernatant (approximately 5–10 times). To pellet any potentially contaminating cell debris or beads, the combined supernatant was centrifuged at 2,500 x g, at 4°C, for 15 min. The resulting supernatant was transferred to 12 mL ultracentrifuge tubes. Soluble and insoluble protein fractions were separated by ultracentrifugation at 100,000 x g, at 4°C, for 1 h.

#### Protein preparation for massspectrometry

Proteins were reduced with dithiothreitol (1:50 ratio of dithiothreitol:protein), alkylated using iodoacetamide (1:10 ratio of iodoacetamide:protein) and Trypsin digested (1:50 ratio of enzyme:protein) as described in Foster et al [70]. Digested peptides were purified and concentrated on C18 STAGE-tips [71] and eluted in 80% acetonitrile, 0.5% acetic acid, and dried in a vacuum concentrator (Eppendorf). Dried peptides were resuspended in 100 mM triethylammonium bicarbonate and labeled via chemical dimethylation using light (CH_2_O, for control treatment), medium (CD_2_O, for low Cu treatment), and heavy (13CD_2_O for low FeCu treatment) isotopologues of formaldehyde as described previously [72]. Labeled samples were finally combined together in equal amounts for STAGE-tip purification and subsequently eluted in 80% acetonitrile, 0.5% acetic acid. Eluted samples were dried and resuspended in 0.5% acetic acid for the TO03 set and 1% TFA for the TO05 set [72] (S2 Fig).

#### Liquid chromatography tandem mass spectrometry–LCMS/MS

TO03’s purified peptides were analyzed on the linear-trapping quadrupole—Orbitrap mass spectrometer (LTQ-Orbitrap Velos; ThermoFisher Scientific) on-line coupled to an Agilent 1290 Series HPLC using a nanospray ionization source (ThermoFisher Scientific) including a 2 cm long, 100 μm-inner diameter fused silica trap column, 50 μm-inner diameter fused silica fritted analytical column and a 20 μm-inner diameter fused silica gold coated spray tip (6 μm-diameter opening, pulled on a P-2000 laser puller from Sutter Instruments, coated on Leica EM SCD005 Super Cool Sputtering Device). The trap column was packed with 5 μm-diameter Aqua C-18 beads (Phenomenex, www.phenomenex.com) while the analytical column was packed with 3 μm-diameter Reprosil-Pur C-18-AQ beads (Dr. Maisch, www.Dr-Maisch.com). Buffer A consisted of 0.5% aqueous acetic acid, and buffer B consisted of 0.5% acetic acid and 80% acetonitrile in water. Samples were run on a gradient method where buffer B was from 10% to 25% over 120 min, from 25% to 60% over 20 min, from 60% to 100% B over 7 min, kept at 100% for 2.5 min and then the column was reconditioned for 20 min with buffer A. The HPLC system included Agilent 1290 series Pump and Autosampler with Thermostat set at 6°C. The sample was loaded on the trap column at 5 μL min^-1^ and the analysis was performed at 0.1 μL min^-1^. The LTQ-Orbitrap was set to acquire a full-range scan at 60,000 resolution from 350 to 1600 Th in the Orbitrap to simultaneously fragment the top fifteen peptide ions by CID in each cycle in the LTQ (minimum intensity 200 counts). Parent ions were then excluded from MS/MS for the next 30 sec. Singly charged ions were excluded since in ESI mode peptides usually carry multiple charges. The Orbitrap was continuously recalibrated using lock-mass function. The mass error measurement was typically within 5 ppm and was not allowed to exceed 10 ppm.

TO05’s purified peptides were analyzed using a quadrupole–time of flight mass spectrometer (Impact II; Bruker Daltonics) on-line coupled to an Easy nano LC 1000 HPLC (ThermoFisher Scientific) using a Captive nanospray ionization source (Bruker Daltonics) including a column set up identical to that for TO03. Buffer A consisted of 0.1% aqueous formic acid, and buffer B consisted of 0.1% formic acid and 80% acetonitrile in water. Samples were run on a gradient method where buffer B was from 5% to 20% over 45 min, from 20% to 40% over 45 min then to 100% over 2 min, held at 100% for 15 min. Re-equilibration back to 5% buffer B was done separately by the LC automatically. The LC thermostat temperature was set at 7°C. The sample was loaded on the trap column at 800 Bar and the analysis was performed at 0.25 μL min^-1^. The Impact II was set to acquire in a data-dependent auto-MS/MS mode fragmenting the 17 most abundant ions (one at the time) after each full-range scan from m/z 200 Th to m/z 2000 Th. The isolation window for MS/MS was 2 to 3 Th depending on parent ion mass to charge ratio. Parent ions were then excluded from MS/MS for the next 0.4 min. Singly charged ions were excluded since in ESI mode peptides usually carry multiple charges. The error of mass measurement was typically within 5 ppm and was not allowed to exceed 10 ppm.

#### Analysis of mass spectrometry data

Analysis of Mass Spectrometry data was performed using MaxQuant 1.5.1.0. Originally, the search was performed against a database comprised of the protein sequences from the source organism (TO05, publicly available) plus common contaminants using the default MaxQuant parameters with match between run and re-quantification options turned on. Only those peptides exceeding the individually calculated 99% confidence limit (as opposed to the average limit for the whole experiment) were considered as accurately identified. Another search was performed with identical search parameters but against a larger database that combined protein sequences from both the TO05 genome and our own TO03 transcriptome (predicted proteins on assembled EST contigs). If not noted otherwise, differential expression data is given from the original search. Tables that include both differential expression data from the original and the EST mapped search are found in the respective supplementary tables. The mass spectrometry proteomics data have been deposited to the ProteomeXchange Consortium via the PRIDE [73] partner repository with the dataset identifier PXD006237.

#### Statistical analysis of proteomic differential expression

As described above (Protein preparation for mass spectrometry), peptides were labeled with different isotopologues of formaldehyde depending on their growth regime (i.e. control, lowCu, lowFeCu). Once the peptides of the three treatments were labeled, they were mixed together in a 1:1:1 ratio and analyzed by LC-MS/MS. The differential expression between proteins is then derived through the ratio of the intensities (area under the curve) of the light, medium and heavy peaks for each peptide (see S2 Fig).

We set the criteria to determine statistical significance in the differential expression of a protein as:

1) an observed differential expression ratio in at least two of the three biological replicates; 2) an average of the ratios >2 for significantly up-regulated, and <0.5 for significantly down-regulated proteins; and 3) a p-value of <0.05 for the z.test, determining significant difference of the average ratios between treatments, taking the variance into account. Additionally, any protein that had a ratio of >10 (up-regulated) or <0.1 (down-regulated) in at least one biological replicate was also considered to be an all-or-nothing response and was included in the ‘significantly changed’ set.

#### Protein annotation

Predicted proteins from both the publicly available genome of TO05 (CCMP 1005) and our transcriptome of TO03 (CCMP 1003) were searched against the JCVI in-house curated database PhyloDB for functional annotation using BlastP. To determine gene localization for proteins involved in photosynthesis, three strategies were followed: 1) sequences of candidate genes were compared to the publicly available chloroplast genomes of *T*. *oceanica* (CCMP 1005) [74] and *T*. *pseudonana* [75], 2) the diatom specific chloroplast targeting sequence software ASAFind [76] was used in conjunction with SignalP [77] to find nuclear encoded, chloroplast targeted proteins, and 3) the remaining proteins were blasted against NCBI to find the closest homologs.

#### Light harvesting complex (LHC)—Tree generation

Blasting of the publicly available genome of TO05 against the JCVI in-house curated database PhyloDB resulted in 68 predicted proteins containing Chl*a* binding sites typical for family members. Alignment with other known heterokont LHCs showed that 48 of these predicted *T*. *oceanica* LHCs had both the expected three trans-membrane helices as well as eight conserved residues [78–80]. For phylogenetic tree analysis, the amino acid sequences of these 48 predicted *T*. *oceanica* gene models were aligned with 33 *T*. *pseudonana* gene models using the multiple sequence alignment algorithm MAFFT [40,41]. Alignments were edited using the software package Bioedit [81] resulting in 81 aligned sequences consisting of 118 characters, including 23 gaps. The phylogenetic tree was inferred using PhyML 3.0 (100 bootstrap replicates) [82]. Branch support was determined with three different methods: SH-aLRT, aBayes and standard bootstrap [83]. The phylogenetic tree was visualized using TreeView [84–86].

## Results

The two strains of the oceanic diatom *T*. *oceanica* studied were both isolated from the Sargasso Sea, twenty-seven years apart (TO05 in 1958, TO03 in 1985). Despite being identical species, they have exceptionally different Cu requirements. The data presented below illustrate the contrasting physiology of these strains in response to Cu limitation. We structure our results in four sections; first, we describe the effects of Cu limitation on whole cell physiology, followed by a more focused description of the effects on photophysiology. We then present a brief overview of the two proteomic datasets. We conclude with a portrayal of the nature and expression patterns of the underlying light harvesting complexes and proteins involved in photosynthesis, responsible for the changes we see in the photophysiological results.

### Effect of chronic copper limitation on whole cell physiological parameters

**Fig 1:**
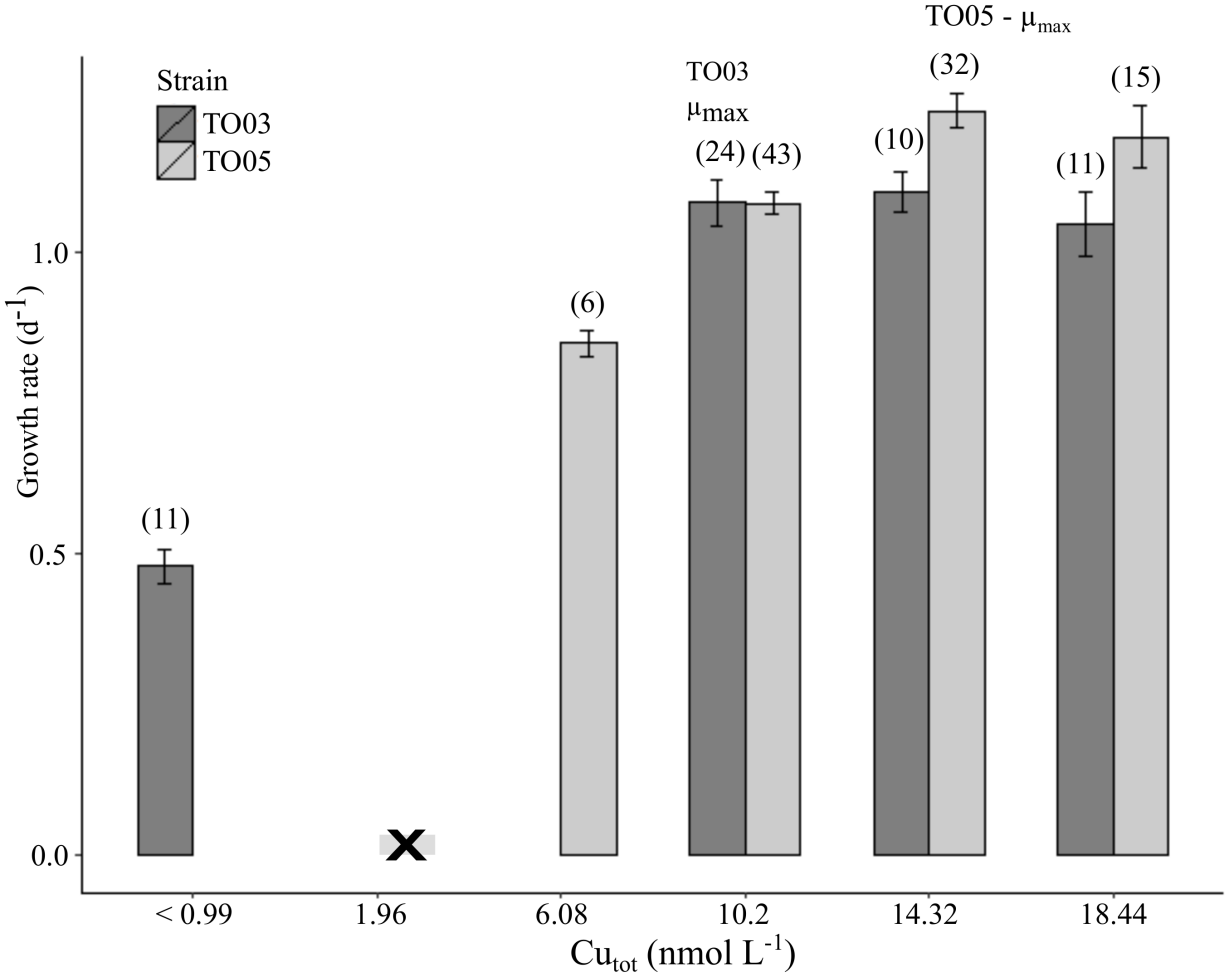
Copper dependent growth rates of *Thalassiosira oceanica* TO03 and TO05. Mean values ± standard error are shown; numbers of biological replicates (n) are indicated in brackets. Note that TO05 was not able to grow under 1.96 nM Cu in the medium (indicated by X in the graph). Given the scope of this study, TO03 was not grown under 1.96 nM or 6.08 nM, as it was able to grow under Cu concentrations <1 nM.

**Fig 2:**
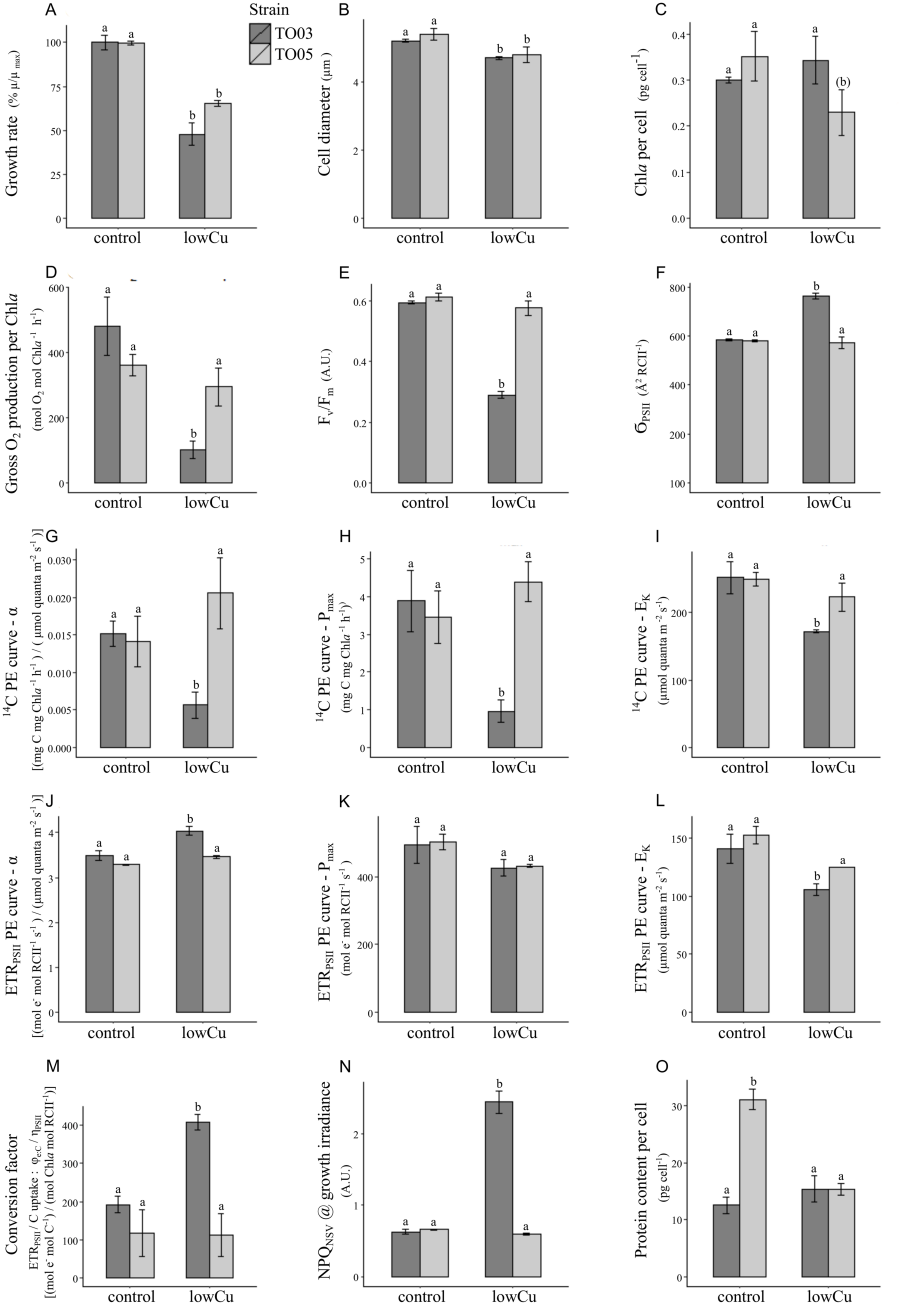
The effects of Cu limitation on growth rate, cell diameter, protein content and a series of photophysiological parameters in two strains of *T*. *oceanica*. **2A-C**, growthrate, cell diameter, and Chl*a* per cell, respectively; **2D-F**, gross oxygen production, Fv/Fm, and the absorption cross section of PS II antennae, respectively; **2G-I**, ^14^C PE curve parameters α, P_max_, and E_K_, respectively; **2J-L**, ETR_PSII_ PE curve parameters α, P_max_, and E_K_, respectively; **2M-O**, conversion factor, NPQ_NSV_, and cellular protein content, respectively. The values are mean ± std. error of three biological replicates. Differing letters above bars represent statistically significant changes (p < 0.05) using a 2-way ANOVA with post-hoc interaction analysis (see Methods for details). Note that both strains have the same physiological responses under metal replete conditions. Under low Cu conditions, only growth rate and cell size are significantly reduced in the same manner in both strains.

TO03 achieved a maximum specific growth rate (μ_max_) of 1.05 per day (d^-1^) in the presence of 10.2 nM [Cu]_total_ (Fig 1). The background Cu concentration in the media (<1 nM [Cu]_total_) allowed TO03 to sustain a growth rate of 0.51 d^-1^, equivalent to 48% of μ_max_ (± 6.45% std error). In contrast, TO05 could not survive at this lowCu level (<1 nM [Cu]_total_) and achieved its maximum growth rate (1. 26 d^-1^) at higher Cu concentrations (14.3 nM [Cu]_total_) than TO03. In TO05, Cu limitation was induced at 6.08 nM [Cu]_total_ (0.82 d^-1^, or 65% μ_max_). Even though the absolute growth rates under Cu replete and deplete conditions are statistically different for the two strains, their relative Cu-limited growth rates (μ/μ_max_) are indistinguishable (p>0.1, Fig 2A, S1 Table). Therefore, comparing the effect of Cu limitation between the strains is appropriate.

Cell diameter decreased 10% in both strains in response to low Cu (Fig 2B, S1 Table). Chl*a* concentration per cell was constant in TO03, while it decreased somewhat in TO05 (Fig 2C). However, as cell diameter decreased in both strains, when normalized to cell volume, Chl*a* concentration increased in response to low Cu in TO03 (4.3±0.1 to 7.4 ±0.5 fg Chl*a* fL^-1^, p<0.001) and stayed the same in TO05 (4.3±0.8 and 3.8 ±0.4 fg Chl*a* fL^-1^, S1 Table). Cellular protein content remained constant in TO03. However, TO05 significantly decreased its cellular protein content in response to low Cu by 50% (Fig 2O). Gross oxygen production decreased in TO03 in response to Cu limitation, regardless of the parameter used to normalize the rates (S1 Table): normalized to Chl*a*, it decreased by 72% (Fig 2D), and normalized to cell, it decreased by 70% (123±11.5 to 36 ±4.3 fmol O_2_ cell^-1^ h^-1^, p<0.001). In contrast, in TO05, O_2_ production was only significantly decreased when normalized to cell (139±10.4 to 69.4 ±2.0 fmol O_2_ cell^-1^ h^-1^, p<0.01, S1 Table) but not when normalized to Chl*a*. Respiration, on the other hand, was not affected by Cu concentration in either of the two strains, independent of normalization (S1 Table).

### Effects of Cu limitation on photophysiology, ETR_RCII_and C-assimilation

Chronic Cu limitation had profound effects on the photophysiology of TO03, but not TO05. In fact, none of the following photophysiological, ETR_RCII_, or C-assimilation parameters changed in TO05 in response to low Cu. In TO03, Cu limitation resulted in a decrease of F_v_/F_m_ from 0.6±0.01 to 0.3±0.01 A.U., p<0.001 (Fig 2E). The functional absorption cross-section of PSII (σ_PSII_) increased by 30% (Fig 2F) and the plastoquinone (PQ) pool size increased by 37% (4.3 ±0.5 to 6.0±0.4 PQ mol Q_B_^-1^, p< 0.1) (S1 Table).

To gain more insight into photophysiological changes, we generated PE curves for short term C-assimilation and ETR_RCII_. From these curves, we derived the respective photosynthetic parameters P_max_, α, and E_k_. For short-term C-assimilation, normalized to Chl*a*, all three parameters decreased in TO03: P_max_ decreased by 75%, α decreased by 63% and E_k_ decreased by 54% (Fig 2G–2I). For ETR_RCII_ normalized to RC (Fig 2J–2L), α increased by 18% in response to low Cu, while P_max_ remained constant and E_k_ decreased by 25%. For both strains, the light saturation point E_k_ was higher for carbon fixation than ETR_RCII_ (Fig 2I and 2L), implying that ETR_RCII_ saturated at lower light intensities than carbon fixation. At the growth irradiance (155 μmol quanta m^-2^ s^-1^), the rates of ETR_RCII_ relative to those of C-assimilation (i.e. the conversion factor φ_e:C_/η_PSII_) increased two-fold in response to low Cu in TO03 (Fig 2M).

In line with the study by Schuback *et al*. [49], we examined FRRF derived parameters at the growth irradiance to further elucidate the photophysiological mechanisms. The following parameters changed only in TO03 and not in TO05. Low Cu decreased the efficiency of excitation energy capture by the fraction of open RCII **(**F_v_′/F_m_′) by 53% in TO03 (0.6±0.01 to 0.3±0.01 A.U., p<0.0001, S1 Table). In line with the decrease in F_v_′/F_m_′, we observed a four-fold increase in non-photochemical quenching, estimated as the normalized Stern Vollmer NPQ_NSV_ (Fig 2N). The overall quantum efficiency of photochemical energy conversion in PSII (F_q_′/F_m_′, φ′_PSII_) decreased by 63% in response to low Cu (0.4±0.03 to 0.2±0.01 A.U., p<0.001, S1 Table). In contrast, photochemical quenching (F_q_′/F_v_′), an indicator of the efficiency of charge separation in functional RCII was only slightly reduced by 17% (0.7 ±0.04 to 0.6±0.02 A.U., p<0.05, S1 Table).

### Overview of the two proteomic datasets for the two strains—Original *vs* EST included

**Fig 3:**
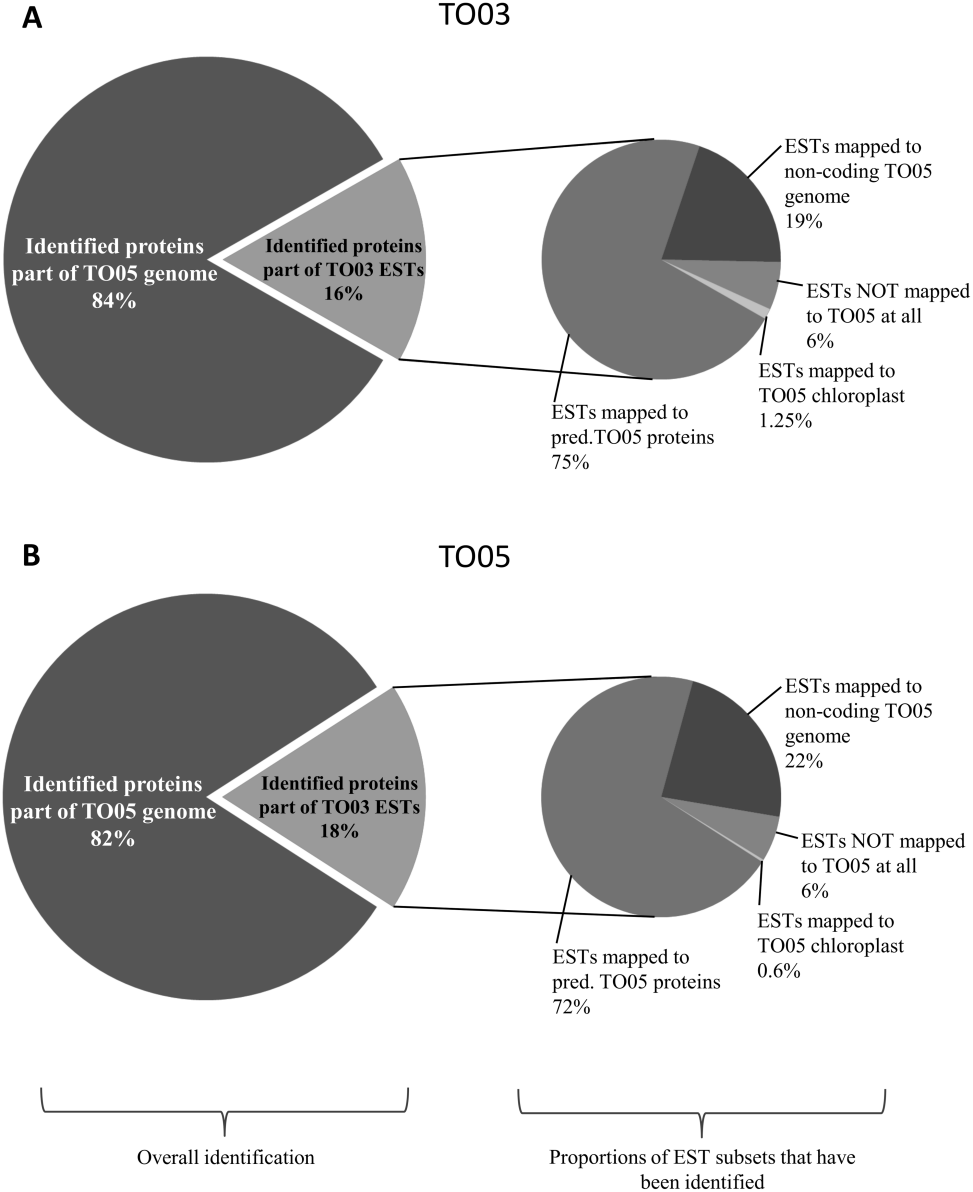
Comparison between EST-mapped proteomics datasets of TO03 and TO05, emphasizing the similarity in their genomes. **A) Results for TO03 (plusEST). B) Results for TO05 (plusEST)**. The left side of the panel shows the proportion of identified predicted proteins coming from the TO05 genome *vs*. the TO03 transcriptome (ESTs). The right side of the panel shows the proportion of the different types of ESTs that have been mapped by the peptides coming from the LC-MS/MS. Even though we used the combined database including all known TO05 and TO03 predicted proteins, neither strain shows bias towards its own subset of proteins.

For each strain, we mapped the peptides generated by the LC-MS/MS to two databases (see Methods). In the original search (thereafter called 'original'), peptides were mapped to proteins from the TO05 genome. To ensure that we did not miss any important information regarding the unique response of TO03 due to possible genomic differences between the two strains, we added the second search (thereafter called 'plusEST'), were the same peptides were mapped to a larger database composed of the original protein sequences of the TO05 genome plus the protein sequences of our own TO03 transcriptome. The plusEST search lead to a moderate increase of identified proteins in both strains when comparing the results with the respective original search (15.6% in TO03 and 13.4% in TO05, see S4 Table). As illustrated in Fig 3, the distribution of the identified proteins in both strains is as follows: approximately 83% of all identified proteins had best hits in the TO05 genome part and 17% (e. g. TO03: 139 ESTs) in the EST part of the database. Examining these 139 hits, we also observe a similar proportion of EST sub-groups represented in both strains: 75% of these ESTs can themselves be mapped to predicted proteins of the TO05 genome, a little over 19% map to putatively non-coding regions of the TO05 genome, and 1.25% map to the chloroplast genome, whereas 6% do not map anywhere on the TO05 genome.

### Proteomic shift in the antennae and photosynthetic transport chain

#### Identity and expression of LHC proteins

**Fig 4:**
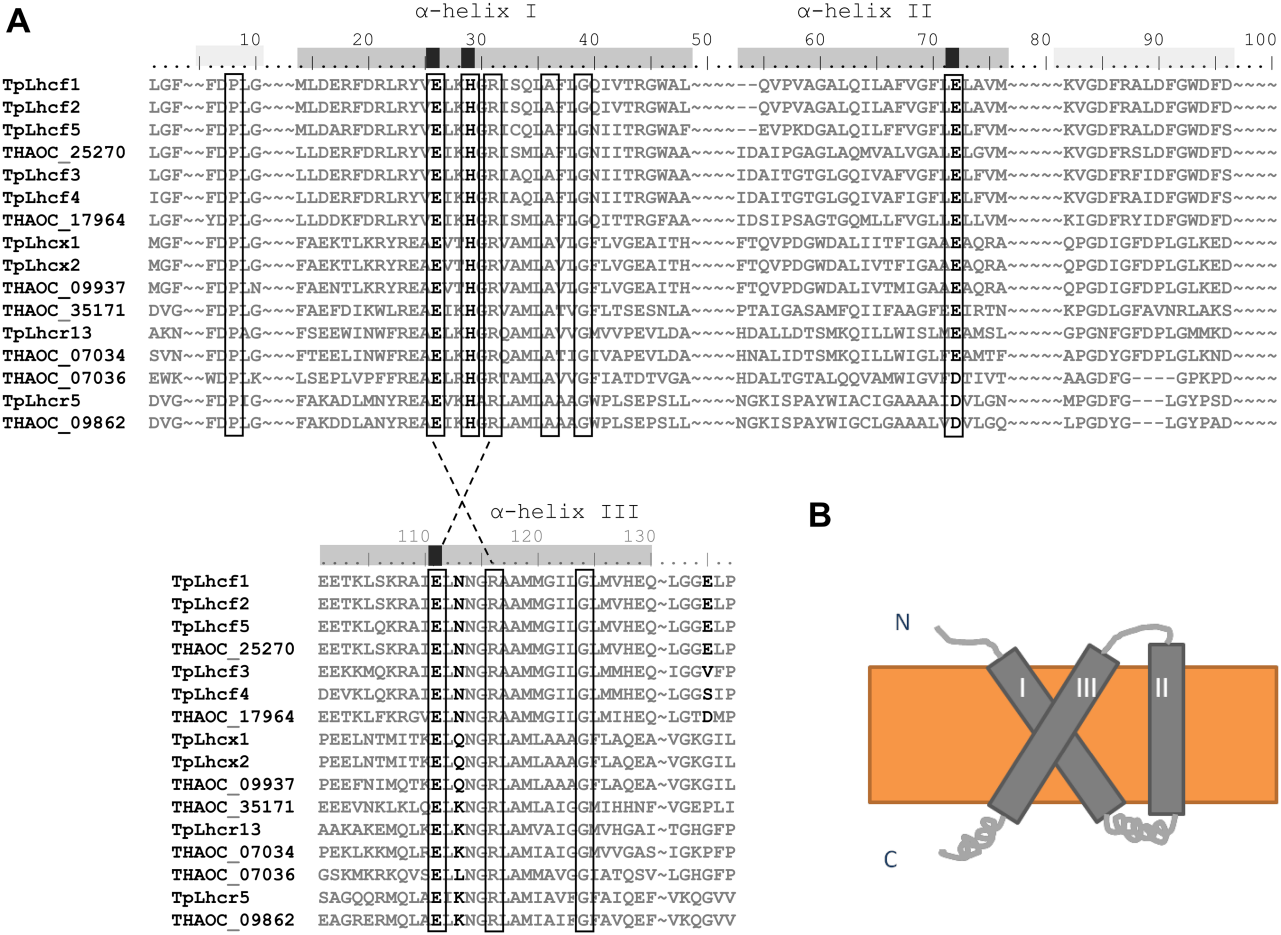
**A, Representative sequence alignment of predicted LHCs from *Thalassiosira oceanica* (THAOC, CCMP 1005) and *T*. *pseudonana* (Tp, CCMP 1335)**: boxes indicate conserved residues; dotted lines show linkage between helix I and III; bold residues are predicted binding sites for Chl molecules. **B, Cartoon of the predicted general LHC structure (grey) comprised of three membrane spanning helices within the thylakoid membrane (orange) and the lumenal N-terminus and stromal C-terminus [**80**]**.

**Fig 5:**
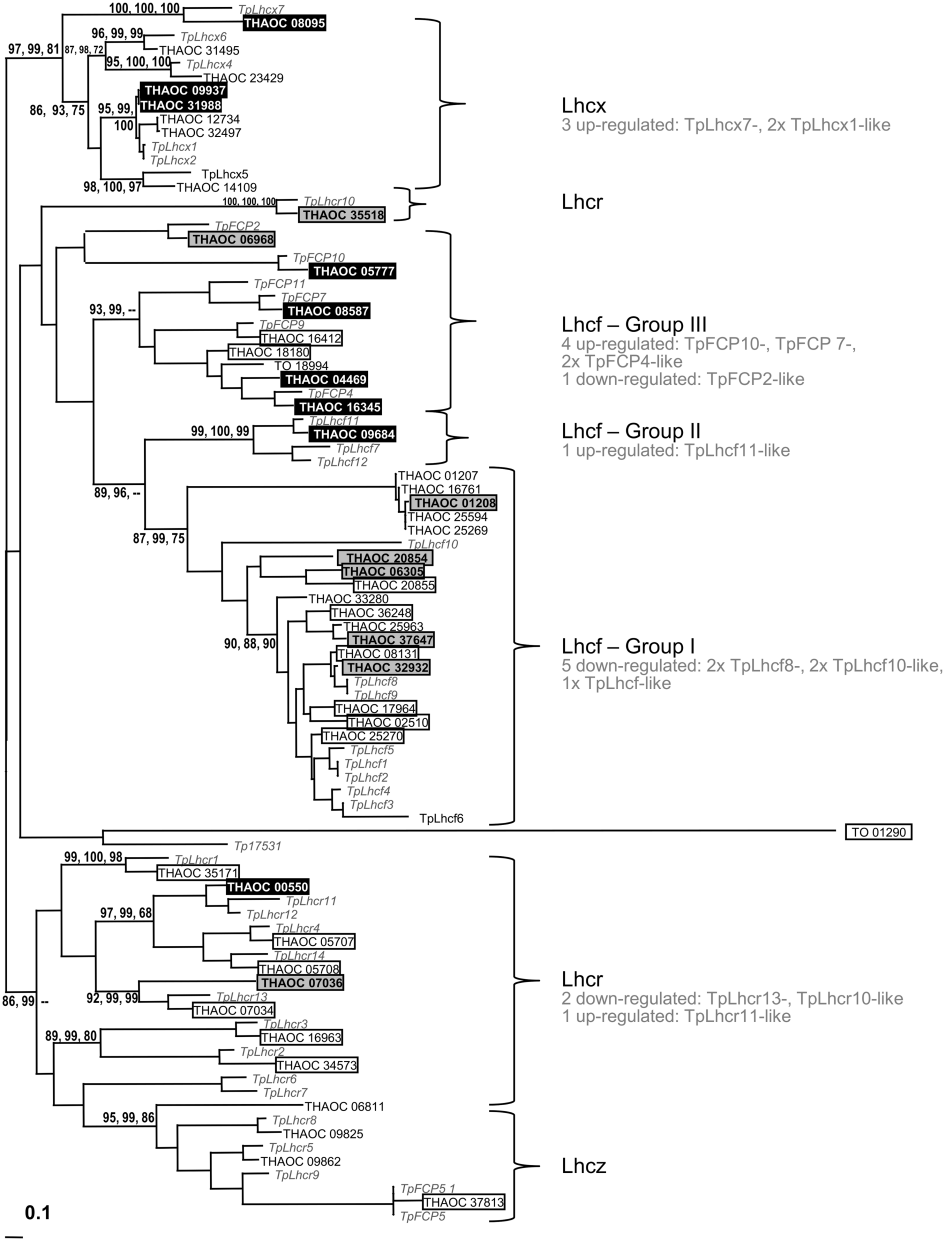
Phylogenetic tree of 48 predicted LHCs from the *T*. *oceanica* genome (CCMP 1005) aligned with 41 LHCs from *T*. *pseudonana* (CCMP 1335). Boxed THAOC LHCs have been identified at the protein level in this study. LHCs in grey shaded boxes with bold, black letters are significantly down-regulated and those in black shaded boxes with bold, white letters are significantly up-regulated in response to low Cu in TO03. The only significantly regulated LHC in TO05 is THAOC_08587 (up), which aligns closest to TpFCP7 (Table 2). Numbers on nodes are based on PhyML alertSH, aBayes, and standard bootstrap (100 replicates) and are expressed as percentages. Bootstrap values below 65% are not shown.

*In silico* probing of the publicly available genome of *Thalassiosira oceanica* (CCMP 1005) for proteins containing a predicted Chl*a/b* binding site, revealed 69 potential candidate genes coding for light harvesting complex (LHC) proteins. Aligning the predicted protein sequences with those of 41 predicted LHCs from the closely related diatom *Thalassiosira pseudonana* (CCMP 1335) revealed that only 48 LHCs from *T*. *oceanica* feature all three helices with conserved residues deemed essential for their proper functioning (Fig 4, S2 Table) [78–80]. Of the 48 predicted proteins in *T*. *oceanica*, 33 LHCs (69%) were identified at the protein level in this study (Fig 5, Tables 1 and 2).The overall expressed inventory of LHCs was very similar between the two strains.

**Table 1:** Overview of expression of all putative LHCs in both strains of *T*. *oceanica*.

clade^a^	predicted	TO03 expressed^b^	TO05 expressed^b^	TO03 up^c^	TO03 down^c^
All	48	30	32	9	8
Lhcf - Group I	17	10	10	-	5
Lhcf - Group II	1	1	1	1	-
Lhcf - Group III	9	7	8	4	1
Lhcr	10	9	9	1	2
Lhcx	8	3	3	3	-
Lhcz	2	-	-	-	-
17531	1	-	1	-	-

^a^clade assignment as per phylogenetic tree (Fig 5)

^b^for full list of expressed LHCs see S2 Table

^c^up/down refers to significant differential expression as defined in methods

**Table 2:** Significantly differentially expressed LHCs under chronic low Cu conditions.

clade^a^	gene name (NCBI)	closest homolog in Tp^a^	TO03 sig regulated^b^	TO05 sig regulated^b^	Evidence for role in other diatoms
Lhcf—Group I	THAOC_06305	TpLhcf	-10.34^c^		light harvesting
	THAOC_20854	TpLhcf	-5.56		light harvesting
	THAOC_01208	TpLhcf	-2.79		
	THAOC_32932	TpLhcf8	-2.12		light harvesting, trimers, oligomers [87]
	THAOC_37647	TpLhcf	-2.04		
Lhcf—Group II	THAOC_09684	TpLhcf11	4.83		
Lhcf—Group III	THAOC_06968	TpFCP2	-2.52		in TP tightly bound to PSI [88]
	THAOC_04469	TpFCP4	2.06		
	THAOC_05777	TpFCP10	2.40		
	THAOC_16345	TpFCP4	2.68		
	THAOC_08587	TpFCP7	2.79	2.07	close to haptophyte LHCs [88]
Lhcr	THAOC_07036	TpLhcr13	-6.57		PS I light harvesting [89–91]
	THAOC_35518	TpLhcr10	-2.55		PS I light harvesting [90,92]
	THAOC_00550	TpLhcr11	2.39^c^		PS I light harvesting [90,92]
Lhcx	THAOC_08095	TpLhcx7	2.76^c^		
	THAOC_09937	TpLhcx1	3.29		photoprotection, stress response, associated with both PS I + II, FCP trimers, oligo, or only loosely associated with membrane, facilitates NPQ [10,90,92–94]
	THAOC_31988	TpLhcx1	3.29		

Tp, *Thalassiosira pseudonana*

^a^as per phylogenetic tree (Fig 5)

^b^significant differential expression in original dataset as defined in methods, given in fold-change

^c^significant differential expression in EST dataset as defined in methods, for Table showing all expressed LHCs and including differential expression in both original and EST datasets, see S2 Table

Of the 48 predicted gene models, we found evidence at the protein level for 33 LHCs (29 found in both, 1 only in TO03, and 3 only in TO05, S2 Table). Note that the number of proteins expressed per LHC clade is similar between the two strains. Considerable numbers of significantly differentially expressed LHCs are only found in TO03 with 9 up- and 7 down-regulated, whereas there is only one up-regulated in TO05 (Group III, see Table 2).

Phylogenetic analysis of these 48 putative LHCs in *T*. *oceanica* (CCMP 1005) led to their classification into the four previously described clades (Fig 5, Tables 1 and 2, S2 Table) [95–97]: the main Chl *a/c* Lhcf (26 predicted proteins), the red algal-like Lhcr (10 predicted proteins), the related clade Lhcz (three predicted proteins), and the LI818-like clade Lhcx (eight predicted proteins). The additional clade around Tp17531 has been added, given that at least 5 different diatoms have a protein that clusters here [96], including *T*. *oceanica* (this study). Furthermore, the Lhcf clade can be divided into three subclades: Lhcf- Group I-III.

Of the 33 LHCs identified at the protein level, 88% (29 LHCs) are expressed in both strains. However, whereas TO05 has only one up-regulated LHC in response to low Cu concentrations (THAOC_08587, Lhcf-group III, TpFCP7 homolog) and none down-regulated, TO03 demonstrates a well-orchestrated response (Fig 5, Table 2). Approximately 55% of its expressed LHCs were significantly regulated in response to Cu limitation. Eight LHCs were down-regulated: four proteins in the Lhcf- Group I, one in the Lhcf–Group III and two in the Lhcr clade. Nine LHCs were up-regulated: one protein in the diatom specific Lhcf- Group II, four in Lhcf—Group III, one in the Lhcr clade and three in the Lhcx clade.

#### Proteomic shift within the chloroplast electron transport chain (ETC)

**Fig 6:**
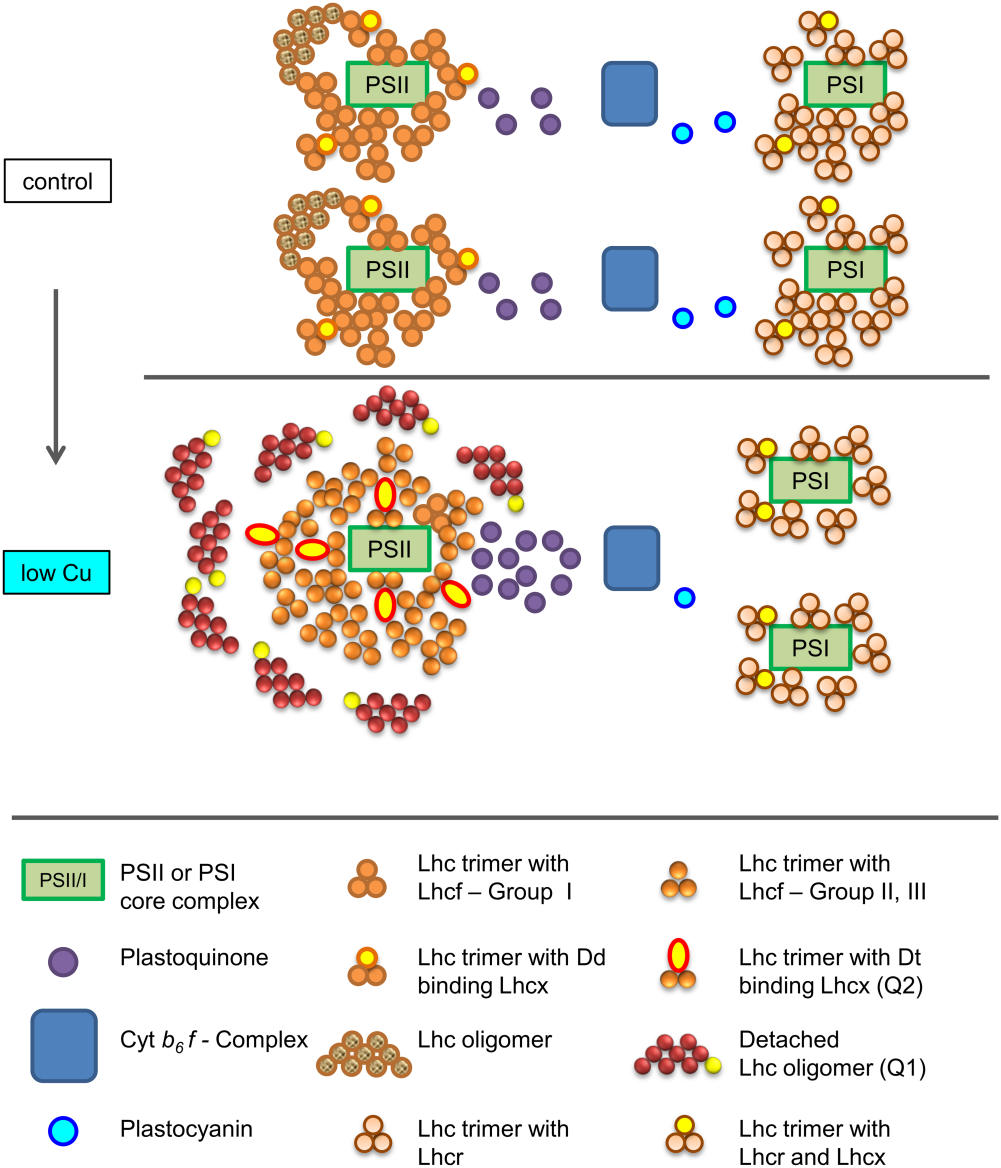
Model of the changes of the photosynthetic apparatus in *T*. *oceanica* (CCMP 1003) in response to growth in the presence of low Cu compared to optimal Cu levels (control). In the process of acclimation to low Cu concentration, several structural rearrangements take place in the photosynthetic apparatus allowing maximal photosynthetic efficiency and minimal photoinhibition. In our model, the represented changes in the numbers of LHCs, protein complexes and electron carriers are a reflection of the changes we observed in the proteomic data (see Table 3 for details): Overall cellular concentrations of proteins associated with PSII, and the photosynthetic electron transport chain (PSII, cyt*b*_*6*_*f* complex and plastocyanin) decrease, whereas those associated with PSI remain unchanged, and the size of the plastoquinone pool increases. In general, cellular Chl*a* concentration stays constant with an increase in the absorption cross section σ_PSII_. NPQ increases dramatically as reflected in both a) quenching sites Q1 and Q2 located in detached oligomers (DLHCs) and b) trimers with diatoxanthin (Dt) binding Lhcx that are bound to PSII, respectively (NPQ response adapted from [97]). Approximate pigment content per LHC monomer is 8 Fucoxanthin, 4–6 Chl*a*, 2 Chl*c* (as per [98]). For more details see “Proteomic shift within the photosynthetic apparatus in TO03” in the discussion.

In both strains, many of the proteins involved in the photosynthetic electron transport chain (ETC) were identified (23 in TO03, 24 in TO05) (Table 3); 17 of these were shared, whereas five and six were only found in TO03 and TO05, respectively. Acclimation to low Cu has a profound impact on the stoichiometry of the complexes involved in the ETC in strain TO03. Here, protein levels of PSII (psbC, psbD, psbE, psbQ), cyt*b*_*6*_*f* (petB), and the Cu-containing electron carrier plastocyanin (petE) are all significantly reduced (2-fold, 2-fold, and 4-fold, respectively) (Table 3). Protein levels for PSI are unchanged in response to low Cu (psaA, psaB, psaC, psaD, psaF) whereas ferredoxin (petF) and FNR (petH) are both induced (40-fold and 2.4-fold, respectively). TO05 shows no significant differential expression of any of the proteins involved in the photosynthetic ETC. The EST mapped data support these findings (see S3 Table). We present a model of the photosynthetic electron transport chain in TO03, visualizing the stoichiometric changes of its major components in response to chronic Cu limitation (Fig 6).

**Table 3:** Differential expression of proteins involved in the photosynthetic electron transport chain (ETC) in response to chronic Cu limitation in *T*. *oceanica*.

Part_of	gene name (NCBI) ^a^	Protein Description^a^	differential expression^a^^,^^b^		where encoded
			TO03	TO05	
PS II	THAOC_34020	psb27-like, involved in Mn cluster formation	1.73	1	Nuc
	psbA	psbA, photosystem II protein D1	-1.92	-1.03	C
	psbB	psbB, photosystem II CP47 reaction center protein	-1.61	-1.1	C
	**psbC, THAOC_26185**	**psbC, photosystem II CP43 reaction center protein**	**-2.1**	1.05	C
	**psbD, THAOC_24371**	**psbD, photosystem II D2 protein**	**-2.1**	-1.01	C
	**psbE, THAOC_24363**	**psbE, cytochrome b559 subunit alpha**	**-2.25**	-1.21	C
	psbH	psbH, photosystem II reaction center protein H		-1.49	C
	THAOC_03193	psbO, Mn-stabilizing protein	-1.41		Nuc
	THAOC_15373	psbP, oxygen-evolving enhancer protein 2 (OEE2)		-1.03	Nuc
	**THAOC_08500**	**psbQ, oxygen-evolving enhancer protein 3 (OEE3)**	**-2.96**		Nuc
	THAOC_09685	psbU-like, small extrinsic protein	1.62	1.32	Nuc
	psbV, THAOC_30541	psbV, cytochrome c-550	1.07	1.18	C
	psbY	psbY, photosystem II protein Y		-1.11	C
PET	petA	petA, cytochrome f	-1.63	-1.11	C
	**petB, THAOC_26188**	**petB, cytochrome b6**	**-2. 33**		C
	THAOC_33417	petC, Fe-S subunit (Rieske protein)	-1.51		Nuc
	petD, THAOC_24366	petD, cytochrome b6-f complex subunit 4		1.1	C
	**THAOC_29732**	**petE, plastocyanin**	**-4.41**	1.31	Nuc
PS I	psaA	psaA, photosystem I P700 chlorophyll a apoprotein A1	-1.21	1.14	C
	psaB	psaB, photosystem I P700 chlorophyll a apoprotein A2	1.11	1.01	C
	psaC	psaC, photosystem I iron-sulfur center	-1.18	1.2	C
	psaD, THAOC_24369	psaD, photosystem I reaction centre subunit II	1.2	1	C
	psaF	psaF, photosystem I reaction centre subunit III	-1.2	1.04	C
	THAOC_24361	psaL, photosystem I reaction centre subunit XI	-1.56	-1.02	C
PET	**THAOC_25559**	**petF, ferredoxin**	**43.79**	-1.1	Nuc
	**THAOC_36724**	**petH, FNR—ferredoxin—NADP+ reductase**	**2.47**		Nuc
	THAOC_06509	petH—FNR—ferredoxin—NADP+ reductase		-1.13	Nuc

PSII, photosystem II; PET, photosynthetic electron transport; PSI, photosystem I, Nuc, nucleus; C, chloroplast

^a^content in bolt indicates significantly differentially expressed proteins in TO03, as defined in methods

^b^differential expression given in fold-change of original dataset; for Table including differential expression in EST dataset, see S3 Table

## Discussion

Low copper (Cu) concentrations in the media elicited a remarkably different cellular response in the two strains of the oceanic diatom *T*. *oceanica* (CCMP 1003 and CCMP 1005). This in itself is striking and will be discussed below. However, the main body of the discussion will focus on the effect of low Cu on TO03 (*T*. *oceanica*, CCMP 1003), showing an extensive restructuring of the photosynthetic apparatus, shifting from a primarily light harvesting to a photoprotective role. This restructuring is reflected in a) the composition of the light harvesting antennae, b) the stoichiometry of components of the photosynthetic electron transport chain, and c) the difference in the ratio of electron transport and carbon fixation rate. Differences between the well documented response to low Fe and that to low Cu presented here are discussed.

### Differences between *T*. *oceanica* CCMP 1003 and CCMP 1005

The two strains of the open ocean diatom *T*. *oceanica*, CCMP 1003 (TO03) and CCMP 1005 (TO05), were isolated from the Sargasso Sea 27 years apart, and are considered the same species based on morphology. In our study, the species identity of the two *T*. *oceanica* strains was confirmed, using comparison of the diatom bar coding region encompassing the ITS1–5.8S –ITS2 [36,37] (S1 Sequence). Further evidence of their close genomic relationship is given when we compare the additional proteomic datasets of TO03 (EST) and TO05 (EST) (Fig 3). In both strains, peptides were mapped to a combined database comprised of predicted protein sequences derived from the TO05 genome and our TO03 transcriptome. We predicted that TO03 would have a higher proportion of identified proteins coming from the mapping of the peptides to its own transcriptome relative to mapping them to the TO05 genome. In contrast, for TO05 we predicted the opposite: higher proportion of identified proteins coming from the mapping of the peptides to its own genome. However, the distribution of identified proteins using the TO05 genome plus TO03 transcriptome database is practically the same in both strains. Furthermore, even the fraction of EST subsets is the same in both strains (Fig 3). Thus, even though these two strains are the same species, have been isolated from the same oceanic region, and have a similar genomic make up, it is striking that their physiological responses to Cu limitation are so different (Figs 1 and 2, S1 Table).

Strain TO03 was able to sustain growth at a Cu concentration ([Cu]_total_) of <1 nM, whereas strain TO05 required 6 times more Cu ([Cu]_total_ = 6.08 nM). Moreover, to achieve maximal growth rate, TO03 required only 10.2 nM [Cu]_total_, while TO05 required 40% more Cu (14.32 nM Cu; Fig 1, S1 Table). Although different Cu concentrations were used for the two strains to reach their respective μ_max_ and Cu-limited growth rate, they achieved identical relative growth rate reduction under low Cu (~55% μ_max_), allowing comparison between their respective physiological and proteomic responses (Fig 2A, S1 Table).

In addition to their matching growth rate reduction (Fig 2A), both also decreased their cell size by 10% (Fig 2B). Cell size reduction is a common strategy in unicellular organisms in response to nutrient limitation. Decreasing cell size increases surface-area-to-volume ratio, allowing a greater number of nutrient transporters at the cell surface, relative to the cellular nutrient demand, which is proportional to cell volume. Furthermore, smaller cells have a thinner surface boundary layer, facilitating the diffusion of nutrients to the cell membrane [20]. Interestingly, of the 23 parameters presented here, TO05 changed only four significantly, whereas TO03 adjusted 18.

Nutrient limitation usually results in chlorosis, a decrease in cellular Chl*a* concentration [99,100]. This common strategy is only seen in TO05 but not in TO03 (Fig 2C). As described in more detail below, TO03 restructured its whole photosynthetic apparatus under low Cu, changing its Chl*a* content (normalized to cell volume, it actually increases, S1 Table), σ_PSII_, F_v_/F_m_, NPQ_NSV_, ETR, and ^14^C uptake. TO05 did not vary any of these parameters (p> 0.05) (Fig 2, S1 Table). Furthermore, of the most prominent cellular Cu-containing proteins (i.e. plastocyanin, cytochrome *c* oxidase), none are down-regulated in TO05.

We speculate that the dominant strategy used by TO05 to deal with Cu limitation is a reduction in cell size (Fig 2B) and overall protein content (Fig 2O). A comparable decrease has been seen in this strain in response to low Fe [13]. An overall reduction in protein content could be expected to result in a decrease in growth rate. However, as the majority of proteins are reduced to the same extent, this would not be detected in our proteomic analysis, because we mixed equal absolute amounts of protein (5 ng control: 5 ng low Cu) independent of changes in cellular protein content. Even though we did not measure Cu quotas, TO05, just by decreasing its overall cellular protein content by 50%, will decrease its Cu-containing proteins by 50%. Strikingly, this agrees well with the 57% decrease in [Cu]_total_ concentration in the media and the resulting 45% decrease in growth rate we observed for TO05.

The gross oxygen production data provide additional evidence for the singular strategy of TO05 to reduce its protein content by 50%. Gross oxygen production, when normalized to cell, decreased by 50% (S1 Table), while when normalized to Chl*a* (Fig 2D) stays constant. This indicates that the photosynthetic unit itself in TO05 was not compromised, while it was greatly affected in TO03. Theoretically, when Cu is extremely low in the medium, the cell would be forced to decrease the proportion of Cu-containing proteins, as we observed for plastocyanin in TO03. However, it seems that TO05 is unable to decrease the Cu-containing proteins (i.e. plastocyanin) relative to other proteins, and thus is unable to grow at extremely low Cu. It is possible that TO05 has lost (or TO03 has gained) a Cu responsive transcription factor able to orchestrate a broad response when Cu concentrations are declining, similar to the one identified in *Chlamydomonas* [101]. TO03 is able to decrease plastocyanin significantly relative to other proteins and has to cope with potentially increased electron pressure and reactive oxygen species. As discussed below, TO03 rearranges its whole photosynthetic apparatus, and consequently, is able to sustain a decent growth rate (~0.5 d^-1^) at extremely low [Cu]_total_ concentrations (<1nM Cu).

In recent years, multiple intra-specific physiological variations have been demonstrated in various phytoplankton taxa, such as the harmful algal bloom (HAB) forming raphidophyte *Heterosigma* [102–104], and diatoms such as *Skeletonema* [105], *Ditylum* [106], *Thalassiosira rotula* [107] and the HAB forming *Pseudo-nitzschia* [108,109]. These intra-specific differences can manifest themselves in spatial and/or temporal distinct occurrences of one strain over the other. However, four distinct strains of *T*. *rotula* co-existed in the North Atlantic spring bloom in 2008 [107]. This co-existence of various strains over large periods of time might facilitate niche-adaptations [110].

However, bearing in mind that diatoms are one of the faster evolving phytoplankton phyla [111], we must still consider the possibility of changes in the genome or epi-genome of organisms kept in long-term culture collections. For example, the green alga *C*. *reinhardtii* took less than 500 generations in nutrient rich media to lose its ability to synthesize cobalamin (Vit B_12_) [112]. To address this issue, we contacted the curator at NCMA; their culture transfer regime (every 2–3 weeks for over >20 years without inverting the tubes) would indeed allow the cells to experience nutrient rich and limiting conditions during each transfer cycle, hopefully preventing changes in genome and epi-genome. In support of this, all the studies investigating trace metal requirements of open ocean and coastal species have used isolates that had been in culture for over 20 years, under trace metal replete conditions. These studies still revealed striking differences in the metal requirements in open ocean and coastal isolates [17,33,113,114]. We believe that the responses of TO05 (i.e. decrease of whole complement of proteome to decrease Cu requirement) and TO03 (i.e. differential expression of proteins) are both viable, but represent very different strategies to cope with Cu limitation.

### Proteomic shift within the photosynthetic apparatus in TO03: Electron transport chain and antennae

#### Electron transport chain—ETC

In the plant photosynthetic electron transport chain (ETC), electrons are channelled from PSII via the PQ pool to cyt*b*_*6*_*f*, then via the small Cu-containing plastocyanin to PSI, from where they are transferred by way of ferredoxin (petF, Fdx) and FNR to NADP+, thereby producing the reducing equivalent NADPH [115]. In addition to the supply of reducing equivalents as NADPH, the ETC generates a proton gradient across the thylakoid membrane ultimately resulting in ATP synthesis. Both ATP and NADPH are needed in the carbon fixing Calvin-Benson-Bassham cycle. Since more ATP is needed for carbon fixation than the amount of ATP that can be generated via the proton gradient generated by this linear electron transport (LET) chain [116,117], additional protons can be pumped into the lumen via cyclic electron transport (CET) either around PSII [118–120] or PSI [121]. Keeping an adequate ATP/ NADPH ratio is imperative for any cell to maximize growth and minimize cell damage. As shown by Bailleul *et al*. [122], in diatoms additional ATP and NADPH can also be supplied to the chloroplast by the mitochondria.

Acclimation to low Cu has a profound impact on the stoichiometry of the complexes involved in the ETC in strain TO03 (Table 3). Changes in ETC stoichiometry have also been observed in TO03 in response to low Fe [9], when Fe-rich complexes, such as PSI and cyt*b*_*6*_*f*, are significantly reduced. However, low Cu concentrations induce a different change in stoichiometry, such that the concentrations of some of the proteins associated with photosystem II, and with the photosynthetic electron transport chain (cyt*b*_*6*_*f*, and plastocyanin) are significantly reduced (Table 3,Fig 6). Protein levels for PSI, however, are unchanged in response to low Cu. The reduction in plastocyanin is expected given that this protein is thought to be the dominant Cu pool in *T*. *oceanica* [15]. Indeed, our proteomic data showed a significant down-regulation of plastocyanin (4-fold)—a mandatory component in the ETC in *T*. *oceanica*—in response to low Cu (Table 3). This is accompanied by the 2-fold reduction of upstream components of the ETC (i.e. cyt*b*_*6*_*f* and PSII), thus avoiding over reduction and ensuing photodamage of PSII. In contrast to the low Fe response, the Fe-rich PSI complex located down-stream of plastocyanin is not affected by Cu limitation. Furthermore, ferredoxin and FNR are both induced under low Cu (Table 3). FNR is up-regulated by 2.4-fold and has been shown to increase the ability to deal with oxidative stress in tobacco plants [123]. Ferredoxin (Fdx, petF) is the most up-regulated protein in our dataset (>40-fold). In the green alga *Chlamydomonas*, one of six different ferredoxin genes is also strongly up-regulated under low Cu and might facilitate chlorophyll biosynthesis [124]. However, Lin *et al*. [125], using transgenic cell lines overexpressing petF, showed an increased ability to deal with reactive oxygen species (ROS) in heat stressed chloroplasts, most likely due to ferredoxin’s ability to donate electrons in ascorbate-mediated ROS scavenging mechanisms. In stark contrast to the proteomic response to low Cu in TO03, TO05 shows no significant differential expression of any of the proteins involved in the photosynthetic ETC (Table 3). Considering that TO03 can grow relatively well at <1nM Cu, while TO05 cannot even survive at 2 nM Cu, our results seem to indicate that TO05 is unable to restructure its photosynthetic apparatus to deal with low Cu.

#### Antennae

Under low Cu, strain TO03 exhibits another striking shift within the light harvesting antennae. While cellular Chl*a* significantly increased when normalized to cell volume (S1 Table), the functional absorption cross section of PSII, σ_PSII_ (Å^2^ RCII^-1^) also increased by 30% (Fig 2F). An increase in σ_PSII_ has also been observed in cultures acclimated to low Fe [49,126], which is believed to compensate for decreased Chl*a* content and/or PSII abundance. The unexpected increase in cellular Chl*a* (p < 0.1) under low Cu could be associated with disconnected LHCs (DLHCs). DLHCs are not energetically connected to photosystems, hence they elevate F_o_ without changing F_m_, thereby decreasing the apparent F_v_/F_m_ (see review [127] and references therein). They may provide a fast pigment pool in the early stages of Fe recovery. Furthermore, DLHCs have been proclaimed to play a vital role in non-photochemical quenching [97,128]. In summary, the proposed presence of DLHCs in TO03 in response to low Cu can explain increased Chl*a* per cell volume (S1 Table), decreased F_v_/F_m_ and increased NPQ_NSV_ (Fig 2E and 2N).

The restructuring of the light harvesting antenna of PSII is further reflected in changes of protein composition in TO03 (Tables 1 and 2). Indeed, the protein abundance of 55% of the 29 LHCs changed significantly under low Cu (Tables 1 and 2). As shown in the phylogenetic tree (Fig 5, summarized in Table 2), the down-regulated proteins are phylogenetically related to known LHCs that are particularly involved in light harvesting reactions in both PSII [87] and PSI [89,90]. Of the 9 up-regulated LHCs, four are part of Lhcf-group III, which is most closely related to the major Lhcf cluster in the haptophyte *Emiliania* [54]. To our knowledge, this group has not been associated with stress response in diatoms in any other study, and was actually down-regulated by high light stress in *Emiliania*. One up-regulated LHC is part of the diatom-specific Lhcf-group II. The most notable set of up-regulated LHCs are the Lhcx homologs. Lhcx1, in particular, has been shown to play an important role in stress responses by maintaining thylakoid membrane stability [91] and supporting the antennas ability for NPQ [10,97,128,129]. This rearrangement supports a general switch within the antennae from light harvesting (for photochemistry) to photoprotection in TO03. This switch is not seen in TO05 which only regulated one LHC (TpFCP7 homolog THAOC_08587: up-regulated).

### PvsE curves (ETR &^14^C), conversion factor

For decades, PE curves have played a central role in elucidating strategies of photoacclimation in different photosynthetically active organisms [130,131]. Various parameters can be derived to obtain a measure of change in photosynthesis (P) per incident light (E, μmol quanta m^-2^ s^-1^). The initial slope of the curve, α, quantifies the linear light-dependent increase of the rate of photosynthesis under sub-saturating light conditions [57]. When irradiance increases beyond the light saturation point, E_k_, photosynthesis starts to become light saturated until the cell reaches its maximum photosynthetic rate P_max_. In our study we generated PE curves for both short-term C assimilation normalized to Chl*a* and electron transport rate in PSII [ETR_RCII_, normalized to reaction centres (RC)]. The two curves respond differently to Cu limitation, demonstrating that low Cu decouples these two rates of photosynthesis.

#### P*vs*E curve—ETR_RCII_ and carbon uptake

At growth irradiance, in response to low Cu, TO03 reduced CO_2_ uptake per chlorophyll by 68%. Furthermore, all PE parameters significantly decreased in response to low Cu: α^14C^ by 63%, E_k_^14C^ by 32%, and P_max_^14C^ by 75% (Fig 2G–2I). This indicates that under the same irradiance, Cu limited cells cannot provide the necessary amount of ATP and/or NADPH to sustain the same rate of carbon fixation as the Cu replete cultures.

Counter-intuitively—considering the 68% decrease in carbon uptake—the rate of charge separation per reaction centre in PSII (ETR_RCII_) is not impacted by low Cu. However, as the number of reaction centres in the cell decreases by 50% under low Cu (as per 2-fold decrease in PSII proteins), the overall cellular ETC performance is impaired. Under Cu limitation, the functional absorption cross section of PSII (σ_PSII_) increased by 30%, whereas PSII concentration decreased 2-fold (Table 3,Fig 6). These changes result in more efficient light harvesting per RCII at low light levels, reflected in both an increase in α^ETR^ and a decrease in the light saturation point E_k_^ETR^ (Fig 2J and 2L). E_k_^ETR^ is reduced to such an extent that cells were effectively growing under light saturating conditions (lowCu E_k_^ETR^ = 105 μmol quanta m^-2^ s^-1^ *vs*. growth irradiance = 155 μmol quanta m^-2^ s^-1^). Maintaining the same maximal rate of charge separation per functional RC, even under severe Cu limitation, assists in preventing accumulation of excess excitation energy which could result in an increase in ROS. Even though protein levels of both PSII and cyt*b*_*6*_*f* decreased to the same extent (~2x), plastocyanin levels decreased even more (>4x) in response to low Cu (Table 3). This disproportionate decrease of a key electron carrier downstream of PSII ultimately restricts the flow of electrons away from PSII. This bottleneck in the linear electron transport chain, if not safely dissipated, could ultimately lead to a built-up of excitation energy and photoinhibition. The increased PQ pool size (mol PQ mol Q_B_^-1^) (S1 Table, Fig 6) is an indication of its needed buffer function. Furthermore, numerous alternative electron sinks have been suggested to alleviate excess excitation pressure. Of these alternative electron sinks, the Mehler reaction [132], photorespiration [133], nitrate reduction [134] and cyclic electron transport around PSI (CET_PSI_) occur after plastocyanin, whereas the short water-water cycle involving plastoquinone terminal oxidase (PTOX) [135,136] channels electrons away from the linear electron transport chain before plastocyanin. Cyclic electron transport around PSII (CET_PSII_) [118–120] could also reduce electron pressure. In CET_PSII_, the e^-^ in RC_PSII_ that has been injected into the ETC, is resupplied by a recycled e^-^ from the reduced plastoquinone pool, thus no additional new e^-^ from the oxygen evolving complex (OEC) is added. This could also explain, in part, the decreased O_2_ production in TO03 in response to low Cu (S1 Table). We identified putative candidate genes involved in these alternative electron flow pathways in the *T*. *oceanica* genome (i.e. the two PTOX homologues THAOC_16363, THAOC_20783). However, none of these were found expressed in any of the four proteomic data sets (2x TO03, 2x TO05). It is possible that these proteins are present but at a concentration below the detection limit of our method. If this is the case in only one of the two treatments, the protein would not be seen in our dataset (see Method section). For example, the abundance of PTOX in a non-stressed cell is as low as 1 per 100 PSII complexes [137].

In summary, up to 50% of the decrease in both carbon uptake and oxygen production in TO03 can be explained by the overall cellular reduction of PSII reaction centres (as seen in the proteomic data), as the ETR in each of those reaction centres is not impaired. The extra reduction (17%) in carbon uptake could be due to the diversion of electrons away from the Calvin-Benson-Bassham cycle as suggested above. Cyclic electron transport around PSII, in particular, could rationalize the additional decrease in oxygen production (extra 5%).

#### Conversion factor

The conversion factor (mol e^-^mol RCII^-1^ / mol C mol Chl*a*^-1^) as a measure of how many charge separations occur per RC_PSII_ in the light reaction of photosynthesis per carbon fixed per Chl*a* in the dark reaction of photosynthesis, has recently been shown to increase in response to low Fe in both natural phytoplankton assemblages and monoclonal *T*. *oceanica* (CCMP1003) cultures [49]. This factor is of particular interest to oceanographers estimating global primary productivity using fluorescence data [49,57]. However, whereas the low Fe-mediated increase is due to an increase in ETR_RCII_ compared to a stable Chl*a* normalized C-assimilation, the low Cu-mediated increase is due to a stable ETR_RCII_ coupled to a substantially decreased Chl*a* normalized C-assimilation rate (Fig 2M, S1 Table). Why is carbon fixation so impaired in TO03, if cellular Chl*a* and rates of initial charge separation (ETR_RCII_) are not affected by Cu limitation? The answer to this question has multiple components: first, the ETR per reaction centre (ETR_RCII_) is not impaired, but given that the overall concentration of PSII decreases 2-fold, overall cellular ETR_RCII_ also decreases; second, higher fraction of absorbed light energy is dissipated as heat, as suggested by a) the 4-fold increase in NPQ_NSV_ (as an estimate of how much excess excitation energy is dissipated as heat, ultimately relieving excitation energy from PSII), b) the 53% decrease in the efficiency of excitation energy capture by the fraction of open RCII (F_v_′/F_m_′) and c) the 63% decrease in overall quantum efficiency of photochemical energy conversion in PSII (Fq′/Fm′, φ′_PSII_, is the fraction of the primary stable electron acceptor Q_A_ in the oxidized state, hence ready to participate directly in ETR) (S1 Table). So even though the abundance of cellular RC_PSII_ is decreased, the cells are able to modulate the amount of excitation energy reaching the functional RC_PSII_ in a way that ETR_RCII_ is not greatly impaired. The slight 17% reduction in photochemical quenching (Fq′/Fv′) indicates a small increase in the reduction level of the photosynthetic electron transport chain, pointing towards the potential of increased ROS production and the need for other electron sinks (as discussed above). In summary, this suggests that TO03 copes with low Cu by dissipating an increased fraction of the absorbed light as heat, thus modulating the amount of excitation energy reaching functional RCII, such that the ETR_RCII_ is not greatly impaired.

## Conclusion

The physiological adaptations of diatoms to cope with Cu limitation are largely unknown. In the present study we investigated the response to Cu limitation in two strains of the model open ocean diatom *T*. *oceanica* (CCMP 1003 and CCMP 1005), focusing on physiological and proteomic changes in the photosynthetic apparatus. Our results show remarkable differences between the adaptations of TO05 and TO03 to low Cu, highlighting significant intra specific variations. In essence, TO03 seems to be able to grow more efficiently under extremely low Cu by decreasing its most abundant Cu-containing protein, plastocyanin, an essential electron carrier between its PSII and PSI. However, decreasing plastocyanin levels promotes a bottleneck in the linear electron transport between PSII and PSI, which increases excitation energy in the pigment antennae and enhances susceptibility to photodamage by the production of ROS. The increased susceptibility to photodamage is counteracted by a) reconstructing the light harvesting antennae (i.e. increasing the ability for photoprotective heat dissipation of absorbed light energy in the pigment antenna), b) decreasing the ETC components prior to plastocyanin (i.e. PSII, cyt*b*_*6*_*f*) to balance the flow of electrons through the ETC, c) increasing the ETC components counteracting ROS (i.e. Fdx, FNR), and d) using other sinks and pathways for excess e^-^ (i.e. CET_PSII_, PTOX, photorespiration). Together, these biochemical shifts explain the observed changes in physiology, such as a) decreased oxygen evolution, carbon fixation, photosynthetic quotient, F_v_/F_m_ and b) increased cellular Chl*a*, sigma, and the conversion factor between ETR_PSII_ and carbon fixation. Therefore, even though the physiological response of TO03 (*e*.*g*. C-fixation rate or growth rate) to low Fe is similar to that of low Cu, this study shows that the underlying restructuring of the photosynthetic apparatus is markedly different for diatoms exposed to low Cu versus low Fe.

One has to be cautious, though, when trying to draw conclusion from one diatom to all. As presented here, a different strain of the same species, TO05, has a vastly different response to low Cu: it reduces its cellular protein content by half, without changing the relative concentration of certain expected proteins, such as the Cu-containing ones. Thus, TO05 seems unable to modulate a concerted response to low Cu, and is unable to grow at Cu concentrations where TO03 thrives.

Strong evidence for Cu limitation of phytoplankton in the ocean is presently lacking. However, based on our data, the concentrations of Cu in some oceanic regions are sufficiently low (e.g.0.5 nM in the North Pacific [138]) to result in sporadic Cu limitation of phytoplankton growth. For example, in regions with Cu concentrations less than 1 nM, large phytoplankton may experience Cu limitation (due to their low surface area to volume ratio). In addition, phytoplankton in Fe limited regions may experience co-limitation by Fe and Cu, as shown by Semeniuk *et al*. [139]. Given our results, similarly to Fe, Cu limitation is likely to negatively affect C export to the ocean interior. Additionally, in response to low Cu, the potentially significant increase in DLHC, which do not participate in energy capture for carbon fixation, might bias global estimates of primary productivity based on satellite derived Chl*a* fluorescence.

## Supporting information

S1 Fig ITS fragment visualization. Approximately700bp long ITS fragment, comprised of ITS1, 5.8SrDNA gene and part of ITS2, 1.5% agarose gel. TO03, *T*. *oceanica* (CCMP1003); TO05, *T*. *oceanica* (CCMP1005); TP, *T*. *pseudonana*; TW, *T*. *weisfloggii*.
(PDF)

S2 Fig Overview of proteomic method. **A) Workflow** First the extracted proteins are trypsin digested. The resulting peptides are then labelled with isotopologues of formaldehyde depending on their growth regime (ctrl = low, green triangle; lowCu = medium, blue triangle; lowFeCu = heavy, red triangle).After peptides of the respective treatments are labelled, they are mixed together in a 1:1:1 ratio and then analyzed together by LC-MS/MS. The differential expression between proteins is then derived through the ratio of the intensities (area under the curve, here depicted as height of bars) of the light (green), medium (blue) and heavy (red) peaks for each peptide. **B) Table of preparation and mixing of samples analyzed by LC-MS/MS** Each biological replicate (three per treatment) is labelled individually. Then one labelled sample of each treatment is mixed together in a 1:1:1 ratio, resulting in three separate biological replicate mixes to be analyzed by LC-MS/MS. Each of these three biological replicate mixes was then analyzed in technical duplicates (TO03) or triplicates (TO05).
(PDF)

S1 Data All physiological data (excel file). (XLSX)

S1 Table Effect of chronic copper limitation on physiology in 2 strains of the open ocean diatom *Thalassiosira oceanica*. CCMP 1003 and CCMP 1005 abbreviated to TO03 and TO05, respectively. Arrows indicate if the response to low Cu statistically increases ↑ or decreases ↓. Shown are means with standard errors for parameters derived from three biological replicates (^ indicates measurements have only been done on two of the three biological triplicates, see raw data in S1 Data). Low Cu results in bold indicate statistically significant differences compared to the respective control treatment. Stars (*, **, ***) indicate the level of significance of a 2-way ANOVA with post-hoc interaction analysis (see Methods for details). The right panel indicates whether the physiological response differs between the two strains; ↓ and ↑ means the result is significantly lower or higher in TO03 compared to TO05. Note that there is hardly any difference in their physiology under their respective replete metal concentrations. However, when Cu limited, roughly half of the tested parameters show significantly different results.
(PDF)

S2 Table Expression of all 48 predicted LHC in TO03 and TO05 across all four datasets. (PDF)

S3 Table Expression of all identified proteins involved in photosynthetic ETC across all datasets. (PDF)

S4 Table Overview of number of identified proteins in the four different proteomic datasets. (PDF)

S1 Sequence Raw ITS region sequence in .phy format. (PHY)
