# Supplementary material for: Contrasting effects of copper limitation on the photosynthetic apparatus in two strains of the open ocean diatom Thalassiosira oceanica
Source: PLoS One. 2017 Aug 24;12(8):e0181753. doi: 10.1371/journal.pone.0181753 (PMC5570362; doi:10.1371/journal.pone.0181753)
Supplement: S1 Fig — Approximately700bp long ITS fragment, comprised of ITS1, 5.8SrDNA gene and part of ITS2, 1.5% agarose gel. TO03, T. oceanica (CCMP1003); TO05, T. oceanica (CCMP1005); TP, T. pseudonana; TW, T. weisfloggii. (PDF) [file pone.0181753.s001.pdf]

Visualization of ITS PCR products

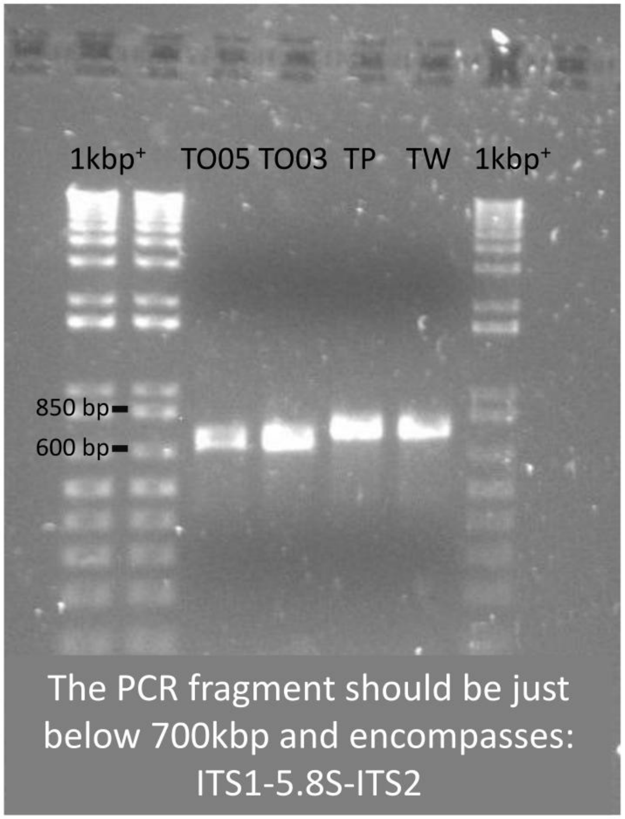

Primers used (White et al, 1990):
ITS1_For: TCCGTAGGTGAACCTGCGG
ITS4_Rev: TCCTCCGCTTATTGATATGC

PCR run (Amato et al, 2007):

Start:	2min	94C
	**35sec**	**94C**
**35 cycles**	**35sec**	**46.2C**
	**60sec**	**72C**
End:	5min	72C

Visualization of PCR products
(2 uL sample + 6x loading buffer, 1 uL kbp+ Ladder Invitrogen):
1.5% Agarose Gel (TAE buffer)
100V

Monitz and Kaczmerska, 2009:
Proposed barcode starts at beginning of 5' end of 5.8s rRNA and ending in the conserved motiv of helix III of ITS-2
"Seven hundred bp of the nuclear internal transcribed spacers (ITS1 and ITS2) and the 5.8S rDNA gene (hereinafter referred collectively as the ITS fragment) were amplified in most cases using primers ITS1 and ITS4"

S1 Fig. Visualization of ITS PCR product. TO05, *Thalassiosira oceanica* (CCMP1005); TO03, *T. oceanica* (CCMP1003); TP, *T. pseudonana*; TW, *T. weissflogii*
