# Supplementary material for: Contrasting effects of copper limitation on the photosynthetic apparatus in two strains of the open ocean diatom Thalassiosira oceanica
Source: PLoS One. 2017 Aug 24;12(8):e0181753. doi: 10.1371/journal.pone.0181753 (PMC5570362; doi:10.1371/journal.pone.0181753)
Supplement: S2 Fig — A) Workflow First the extracted proteins are trypsin digested. The resulting peptides are then labelled with isotopologues of formaldehyde depending on their growth regime (ctrl = low, green triangle; lowCu = medium, blue triangle; lowFeCu = heavy, red triangle).After peptides of the respective treatments are labelled, they are mixed together in a 1:1:1 ratio and then analyzed together by LC-MS/MS. The differential expression between proteins is then derived through the ratio of the intensities (area under the curve, here depicted as height of bars) of the light (green), medium (blue) and heavy (red) peaks for each peptide. B) Table of preparation and mixing of samples analyzed by LC-MS/MS Each biological replicate (three per treatment) is labelled individually. Then one labelled sample of each treatment is mixed together in a 1:1:1 ratio, resulting in three separate biological replicate mixes to be analyzed by LC-MS/MS. Each of these three biological replicate mixes was then analyzed in technical duplicates (TO03) or triplicates (TO05). (PDF) [file pone.0181753.s002.pdf]

A

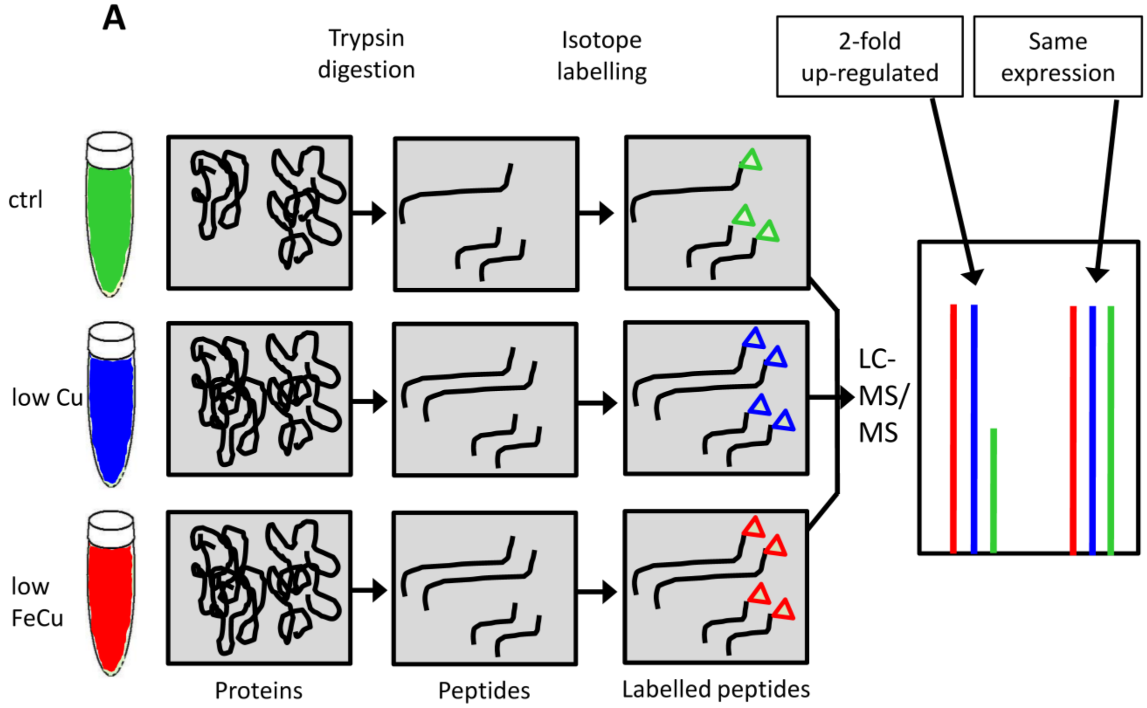

B

Treatment (label)	Biological Replicate		
**Control (low)**	1	2	3
**lowCu (medium)**	1	2	3
**lowFeCu (heavy)**	1	2	3
	↓	↓	↓
1:1:1 mix of labelled samples	1	2	3

S2 Fig. Overview of Proteomic Method. A) Workflow First the extracted proteins are trypsin digested. The resulting peptides are then labelled with isotopologues of formaldehyde depending on their growth regime (ctrl = low, green triangle; lowCu = medium, blue triangle; lowFeCu = heavy, red triangle). After peptides of the respective treatments are labelled, they are mixed together in a 1:1:1 ratio and then analyzed together by LC-MS/MS. The differential expression between proteins is then derived through the ratio of the intensities (area under the curve, here depicted as height of bars) of the light (green), medium (blue) and heavy (red) peaks for each peptide. **B) Table of preparation and mixing of samples analyzed by LC-MS/MS** Each biological replicate (three per treatment) is labelled individually. Then one labelled sample of each treatment is mixed together in a 1:1:1 ratio, resulting in three separate biological replicate mixes to be analyzed by LC-MS/MS. Each of these three biological replicate mixes was then analyzed in technical duplicates (TO03) or triplicates (TO05).
