# Supplementary material for: Contrasting effects of copper limitation on the photosynthetic apparatus in two strains of the open ocean diatom Thalassiosira oceanica
Source: PLoS One. 2017 Aug 24;12(8):e0181753. doi: 10.1371/journal.pone.0181753 (PMC5570362; doi:10.1371/journal.pone.0181753)
Supplement: S1 Table — CCMP 1003 and CCMP 1005 abbreviated to TO03 and TO05, respectively. Arrows indicate if the response to low Cu statistically increases ↑ or decreases ↓. Shown are means with standard errors for parameters derived from three biological replicates (^ indicates measurements have only been done on two of the three biological triplicates, see raw data in S1 Data). Low Cu results in bold indicate statistically significant differences compared to the respective control treatment. Stars (*, **, ***) indicate the level of significance of a 2-way ANOVA with post-hoc interaction analysis (see Methods for details). The right panel indicates whether the physiological response differs between the two strains; ↓ and ↑ means the result is significantly lower or higher in TO03 compared to TO05. Note that there is hardly any difference in their physiology under their respective replete metal concentrations. However, when Cu limited, roughly half of the tested parameters show significantly different results. (PDF) [file pone.0181753.s004.pdf]

Parameter tested	TO 1003 control	TO 1003 low Cu	TO 1005 control	TO 1005 low Cu	TO03 / TO05 control	TO03 / TO05 lowCu
[Fe] (nmol)	1370.00	1370.00	1370.00	1370.00		
[Cu] (nmol)	10.20	0.20	14.32	6.08		
µ (dd-1)	1.52 ± 0.06	↓ 0.73 ± 0.10 ***	1.82 ± 0.02	↓ 1.19 ± 0.03 ***	↓	↓
µ (d-1)	1.05 ± 0.07	↓ 0.51 ± 0.10***	1.26 ± 0.02	↓ 0.82 ± 0.03***	↓	↓
µ (% µmax)	100.50 ± 4.21	↓ 47.86 ± 6.45 ****	99.82 ± 1.19	↓ 65.46 ± 1.61 ****		
cell diameter (µm)	5.22 ± 0.05	↓ 4.72 ± 0.04 ****	5.42 ± 0.16	↓ 4.82 ± 0.23 ****		
cell vol (fl)	74.68 ± 1.93	↓ 55.16 ± 1.43 ****	83.69 ± 7.74	↓ 59.54 ± 8.78 ****		.
cell surface area (µm^2)	85.73 ± 1.52	↓ 70.1 ± 1.23 **	92.34 ± 5.65	↓ 73.31 ± 7.13 **		
cell SA / V (µm^2 / fL)	1.15 ± 0.01	↑ 1.27 ± 0.01 **	1.11 ± 0.03	↑ 1.25 ± 0.06 **		
Chl a conc (pg/cell)	0.30 ± 0.01	0.34 ± 0.05	0.35 ± 0.05	0.23 ± 0.05 .		.
Chl a conc (fg/fl) cell vol	4.34 ± 0.11	↑ 7.40 ± 0.46 ***	4.28 ± 0.80	3.79 ± 0.41		↑
Fv/Fm (A.U.)	0.60 ± 0.01^	↓ 0.29 ± 0.01 ****	0.61 ± 0.01	0.58 ± 0.02 .	↓	↓
Sigma (Å^2 / RCII)	584.40 ± 4.95^	↑ 764.01 ± 12.50 ***	579.15 ± 3.58^	572.76 ± 24.94^		↑
PQ-Pool size (mol PQ / mol Qb)	4.34 ± 0.47^	5.98 ± 0.35 .	5.65 ± 0.18^	5.12 ± 0.19^		
sigma ' @ growth irradiance (Å^2 / RCII)	583.09 ± 18.30	↑ 664.04 ± 10.95 **	585 ± 3.23^	575.61 ± 2.47^		↑
Fq'/Fv' @ growth irradiance (A.U.)	0.7 ± 0.04	↓ 0.58 ± 0.02 *	0.69 ± 0.02^	0.66 ± 0.01^		
Fv'/Fm' @ growth irradiance (A.U.)	0.61 ± 0.01	↓ 0.29 ± 0.01 ****	0.60 ± 0.01^	0.62 ± 0.00^		↓
Fq'/Fm' @ growth irradiance (A.U.)	0.43 ± 0.03	↓ 0.16 ± 0.01 ***	0.41 ± 0.01^	0.41 ± 0.01^		↓
ETR @ growth irradiance (mol e-/RCII * s)	398.33 ± 31.51	373.65 ± 16.77	390.88 ± 7.34^	368.45 ± 5.97^		
NPQ (nsv) @ growth irradiance	0.63 ± 0.03	↑ 2.43 ± 0.15 ****	0.66 ± 0.01^	0.59 ± 0.01^		↑
ETR - PE curve - alpha (mol e-/RCII)/(µmol quanta / m^2 * s)	3.49 ± 0.11	↑ 4.04 ± 0.11 *	3.29 ± 0.01^	3.46 ± 0.03^		↑
ETR - PE curve - pmax pmax (mol e-/RCII * s)	493.72 ± 54.98	425.67 ± 23.75	502.11 ± 23.46^	431.17 ± 4.69^		
ETR - PE curve - ek (µmol quanta / m^2 * s)	140.79 ± 12.48	↓ 105.34 ± 5.07 *	152.55 ± 7.65^	124.6 ± 0.031^		
Conversion factor (ETR / 14C uptake)	192.24 ± 22.23	↑ 406.85 ± 20.43 *	117.55 ± 61.89^	111.99 ± 56.03^		↑
14C - PvsE - curve - α (g C / g Chla * h)/(µmol quanta / m^2 * s)	0.0151 ± 1.69E-03	↓ 5.66E-03 ± 1.75E-03 *	1.41E-02 ± 3.32E-03	2.06E-02 ± 4.70E-03		↓
14C - PvsE - curve - eK (µmol quanta / m^2 * s)	251.97 ± 23.73	↓ 172.00 ± 2.51 **	249.05 ± 10.46	223.04 ± 20.76		↓
14C - PvsE - curve - Pmax (g C / g Chla * h)	3.89 ± 0.81	↓ 0.97 ± 0.29 **	3.46 ± 0.70	4.40 ± 0.54		↓
14C uptake @ 155 uEinstein (g C / g Chla * h)	2.09 ± 0.27	↓ 0.70 ± 0.21 *	1.94 ± 0.44	2.71 ± 0.53		↓
FeDFB uptake (zmol / µm^2 * h)	4.23 ± 1.62	1.03 ± 0.24	5.47 ± 2.83	2.01 ± 2.00 .		
Gross O2 Prod (mol O2 / mol Chla * h)	366.59 ± 28.16	↓ 101.02 ± 26.53***	362.04 ± 33.22	295.11 ± 58.21		.
Gross O2 Prod (µmol O2 / cell * h)	1.23E-07 ± 1.15E-08	↓ 3.60E-08 ± 4.27E-09****	1.39E-07 ± 1.04E-08	↓ 6.94E-08 ± 1.99E-09**		
Respiration (mol O2 / mol Chla * h)	0.07 ± 0.03	0.04 ± 0.01	0.05 ± 0.01	0.06 ± 0.00		.
Respiration (µmol O2 / cell * h)	1.16E-08 ± 2.32E-9	9.10E-09 ± 1.16E-9	1.79E-08 ± 3.79E-9	1.60E-08 ± 3.76E-9		
14C uptake @ 155 uE (mol C / mol Chla * h)	0.16 ± 0.03	↓ 0.05 ± 0.02*	0.15 ± 0.04	0.20 ± 0.04		
Protein content (pg/cell)	12.61 ± 1.47	15.44 ± 2.32	31.15 ± 2.18	↓ 15.36 ± 1.22****	↑	
AOX activity	48.84 ± 0.9	53.60 ± 7.05	60.56 ± 6.23	63.69 ± 7.12		

. p-val < 0.1

* p-val < 0.05

** p-val < 0.01

*** p-val < 0.001

**** p-val < 0.0001

S1 Table Effect of chronic copper limitation on physiology in 2 strains of the open ocean diatom *Thalassiosira oceanica* : CCMP 1003 and CCMP 1005 abbreviated to TO03 and TO05, respectively. Arrows indicate if the response to low Cu statistically increases ↑ or decreases ↓. Shown are means with standard errors for parameters derived from three biological replicates (^ indicates measurements have only been done on two of the three biological triplicates, see raw data). Low copper results in bold indicate statistically significant differences compared to the respective control treatment. Stars (*, **, ***) indicate the level of significance of a 2-way ANOVA with post-hoc interaction analysis (see methods for details). The right panel indicates whether the physiological response differs between the two strains; ↓ and ↑ means the result is significantly lower or higher in TO03 compared to TO05. Note that there is hardly any difference in their physiology under their respective replete metal concentrations. However, when copper limited, half of the tested parameters show significantly different results
