# Supplementary material for: Contrasting effects of copper limitation on the photosynthetic apparatus in two strains of the open ocean diatom Thalassiosira oceanica
Source: PLoS One. 2017 Aug 24;12(8):e0181753. doi: 10.1371/journal.pone.0181753 (PMC5570362; doi:10.1371/journal.pone.0181753)
Supplement: S2 Table — (PDF) [file pone.0181753.s005.pdf]

S2 Table. Expression of all predicted LHC in TO03 and TO05 across all four datasets.

				differential expression[h)]							
clade[a]	gene name (NCBI)[b]	closest homolog in Tp[c]	expr[d]	TO03 I (original)[e]	sig[g]	TO03 II (+EST)[f]	sig[g]	TO05 I (original)[e]	sig[g]	TO05 II (+EST)[f]	sig[g]
17531	THAOC_01290	**Tp17531**	y					-1.06		-1.05	
Lhcf - Group I	THAOC_02510	**TpLhcf**	y	-1.18		-1.20		-1.13		-1.17	
	**THAOC_06305**	**TpLhcf**	y	-4.2		**-10.34**	y	-1.21		-1.23	
	THAOC_17964	**TpLhcf**	y	1.19		1.06		-1.07		-1.10	
	THAOC_17964, contig_83951_1_204_+	**TpLhcf**	y							-1.07	
	**THAOC_20854**	**TpLhcf**	y	**-5.56**	y	**-5.56**	y	-1.28		-1.27	
	THAOC_20855	**TpLhcf**	y	-1.63		-1.63		-1.21		-1.20	
	THAOC_25270	**TpLhcf**	y	1.23		1.24		-1.13		-1.06	
	THAOC_25270, contig_119270_60_376_-	**TpLhcf**	y							-1.32	
	THAOC_25270, contig_123884_1_219_-	**TpLhcf**	y			-1.70					
	THAOC_25270, contig_70472_60_300_-	**TpLhcf**	y			1.52				-1.07	
	THAOC_25963	TpLhcf									
	THAOC_33280	TpLhcf									
	THAOC_36248	**TpLhcf**	y	-1.38		-1.31		-1.09		-1.12	
	**THAOC_37647**	**TpLhcf**	y.	**-2.04**	y						
	THAOC_08131	**TpLhcf8**	y					-1.32		-1.31	
	THAOC_08131, contig_91916_1_273_+	**TpLhcf8**	y							1.04	
	**THAOC_32932**	**TpLhcf8**	y	**-2.12**	y	**-2.16**	y	-1.15		-1.15	
	THAOC_01207	TpLhcf									
	**THAOC_01208**	**TpLhcf**	y	**-2.79**	y	**-2.81**	y	-1.13		-1.10	
	THAOC_16761	TpLhcf									
	THAOC_25269	TpLhcf									
	THAOC_25594	TpLhcf									
Lhcf - Group II	**THAOC_09684**	**TpLhcf11**	y	**4.83**	y	**3.89**	y	-1.23		-1.24	
Lhcf - Group III	**THAOC_05777**	**TpFCP10**	y	**2.40**	y	**2.32**	y	-1.16		-1.16	
	THAOC_18180	**TpFCP11**	y	2.02				1.09		-1.16	
	**THAOC_06968**	**TpFCP2**	y	**-2.52**	y	**-2.52**	y	-1.09		-1.10	
	**THAOC_04469**	**TpFCP4**	y	**2.06**	y	2.16		-1.26		-1.24	
	**THAOC_16345**	**TpFCP4**	y	**2.68**	y	**2.68**	y	-1.30		-1.29	
	THAOC_18994	TpFCP4									
	**THAOC_08587**	**TpFCP7**	y	**2.79**	y	2.90		**2.07**	y	2.00	
	THAOC_16412	**TpFCP9**	y	1.97		1.97		-1.09		-1.09	
Lhcr	THAOC_06811	TpLhcr									
	THAOC_35171	**TpLhcr1**	y	3.48		3.48		-1.39		-1.37	
	**THAOC_35518**	**TpLhcr10**	y	**-2.55**	y	**-2.56**	y	-1.04		1.03	
	**THAOC_00550**	**TpLhcr11**	y	2.75		**2.39**	y	-1.14		-1.19	
	THAOC_07034	**TpLhcr13**	y	1.66		1.63		-1.11		-1.12	
	**THAOC_07036**	**TpLhcr13**	y	**-6.57**	y	**-6.32**	y	-1.19		-1.12	
	THAOC_05708	**TpLhcr14**	y	-1.06		1.04		1.13		1.12	
	THAOC_34573	**TpLhcr2**	y	4.44		4.42		-1.32		-1.44	
	THAOC_16963	**TpLhcr3**	y	-1.56		-1.45		-1.13		-1.14	
	THAOC_05707	**TpLhcr4**	y	1.24		1.24		-1.21		-1.15	
Lhcx	**THAOC_09937**	**TpLhcx1**	y	**3.29**	y			-1.222			
	THAOC_12734	TpLhcx1									
	THAOC_32497	TpLhcx1									
	**THAOC_31988**	**TpLhcx1**	y			**3.29**	y			-1.22	
	THAOC_23429	TpLhcx4									
	THAOC_14109	TpLhcx5									
	THAOC_31495	TpLhcx6									
	**THAOC_08095**	**TpLhcx7**	y	1.14		**2.76**	y	-1.15		-1.13	
	THAOC_08095, contig_119183_41_788_+	**TpLhcx7**	y			-1.41					
Lhcz	THAOC_09862	TpLhcr5									
	THAOC_09825	TpLhcr8									
	THAOC_37813	**TpFCP5**	y					-1.46		-1.44	

Tp, *Thalassiosira pseudonana*; expr, expressed; sig, significant differential expression

[a]clades as per phylogenetic tree in Figure 4
[b]content in bolt indicates significantly differentially expressed proteins in TO03, as defined in methods; blue, significantly down-regulated; red, significantly up-regulated
[c]bolt names indicate proteins are found expressed in at least one dataset
[d]"y" indicates proteins are found expressed in at least one dataset
[e]original dataset, peptides from LC-MS/MS were mapped against database of predicted proteins of TO05 genome,
[f]EST dataset, peptides from LC-MS/MS were mapped against predicted proteins of combined database consisting of both TO05 genome and our own TO03 Transcriptome (assembled EST contigs)
[g]"y" indicates that the differential expression in the column left of it is significantly regulated as per methods
[h]differential expression given in fold-change, bolt content indicates significantly regulated as per methods
