# Supplementary material for: Contrasting effects of copper limitation on the photosynthetic apparatus in two strains of the open ocean diatom Thalassiosira oceanica
Source: PLoS One. 2017 Aug 24;12(8):e0181753. doi: 10.1371/journal.pone.0181753 (PMC5570362; doi:10.1371/journal.pone.0181753)
Supplement: S3 Table — (PDF) [file pone.0181753.s006.pdf]

S3 Table. Expression of all identified proteins involved in photosynthetic ETC across all datasets.

			differential expression			differential expression	
Part_of	gene name (NCBI)[a]	Protein Description[a]	TO03 I (original)[a,b]	TO05 I (original)[a,b]	where encoded	TO03 II (EST)[a,c]	TO05 II (EST)[c]
PS II	THAOC_34020	psb27-like, involved in Mn cluster formation	1.73	1	Nuc	1.73	1.05
	psbA	psbA, photosystem II protein D1	-1.92	-1.03	C	-1.63	1.07
	psbB	psbB, photosystem II CP47 reaction center protein	-1.61	-1.1	C	-1.65	-1.09
	**psbC, THAOC_26185**	**psbC, photosystem II CP43 reaction center protein**	**-2.1**	1.05	C		1.01
	**psbD, THAOC_24371**	**psbD, photosystem II D2 protein**	**-2.1**	-1.01	C	**-2.01**	
	**psbE, THAOC_24363**	**psbE, cytochrome b559 subunit alpha**	**-2.25**	-1.21	C	**-2.27**	-1.20
	psbH	psbH, photosystem II reaction center protein H		-1.49	C		-1.56
	THAOC_03193	psbO, Mn-stabilizing protein	-1.41		Nuc	-1.40	1.04
	THAOC_15373	psbP, oxygen-evolving enhancer protein 2 (OEE2)		-1.03	Nuc		1.04
	**THAOC_08500**	**psbQ, oxygen-evolving enhancer protein 3 (OEE3)**	**-2.96**		Nuc	-3.94	1.37
	THAOC_09685	psbU-like, small extrinsic protein	1.62	1.32	Nuc	1.63	1.32
	psbV, THAOC_30541	psbV, cytochrome c-550	1.07	1.18	C	1.03	1.18
	psbY	psbY, photosystem II protein Y		-1.11	C		-1.18
PET	petA	petA, cytochrome f	-1.63	-1.11	C	-1.65	
	**petB, THAOC_26188**	**petB, cytochrome b6**	**-2.33**		C	**-2.33**	-1.14
	THAOC_33417	petC, Fe-S subunit (Rieske protein)	-1.51		Nuc		-1.48
	petD, THAOC_24366	petD, cytochrome b6-f complex subunit 4		1.1	C		-1.15
	**THAOC_29732**	**petE, plastocyanin**	**-4.41**	1.31	Nuc	**-4.71**	1.52
PS I	psaA	psaA, photosystem I P700 chlorophyll a apoprotein A1	-1.21	1.14	C	-1.17	1.09
	psaB	psaB, photosystem I P700 chlorophyll a apoprotein A2	1.11	1.01	C	1.46	1.02
	psaC	psaC, photosystem I iron-sulfur center	-1.18	1.2	C	1.15	
	psaD, THAOC_24369	psaD, photosystem I reaction centre subunit II	1.2	1	C	1.20	-1.02
	psaF	psaF, photosystem I reaction centre subunit III	-1.2	1.04	C	-1.14	1.01
	THAOC_24361	psaL, photosystem I reaction centre subunit XI	-1.56	-1.02	C	-2.08	1.31
PET	**THAOC_25559**	**petF, ferredoxin**	**43.79**	-1.1	Nuc	**43.79**	-2.52
	**THAOC_36724**	**petH, FNR - ferredoxin--NADP+ reductase**	**2.47**		Nuc	**2.44**	-1.09
	**contig_64183_1_637_+ (maps to THAOC_36724)**	**petH, FNR - ferredoxin--NADP+ reductase**				**5.50**	
	THAOC_06509	petH - FNR - ferredoxin--NADP+ reductase		-1.13	Nuc	-1.49	-1.17

a) content in bolt indicates significantly differentially expressed proteins in TO03, as defined in methods

b) significant differential expression in original dataset as defined in methods, given in fold-change

c) significant differential expression in EST dataset as defined in methods, given in fold-change
