# Supplementary material for: Contrasting effects of copper limitation on the photosynthetic apparatus in two strains of the open ocean diatom Thalassiosira oceanica
Source: PLoS One. 2017 Aug 24;12(8):e0181753. doi: 10.1371/journal.pone.0181753 (PMC5570362; doi:10.1371/journal.pone.0181753)
Supplement: S4 Table — (PDF) [file pone.0181753.s007.pdf]

S4 Table: Overview of number of identified proteins in the four different proteomic datasets.

	TO03 (original)[e]	TO03 (EST)[f]	TO03 II (%)	TO05 (original)[e]	TO05 (EST)[f]	TO05 II (%)	Diff TO03 (EST and original)	%	Diff TO05 (EST) and (original)	%
peptides identified by LC-MS/MS[a]	4,055	4,055		8,967	8,967					
proteins identified[b]	730	**844**	100%	1431	**1622**	100%	114	16 %	191	13 %
proteins mapped to TO05 genome[c]		**705**	84%		**1327**	82%				
proteins mapped to TO03 EST[d]		**139**	16%		**295**	18%				

proteins mapped to TO03 EST[d]		**139**	100%		**295**	100%
TO03 ESTs mapped to TO05 chloroplast proteins		**2**	1%		**1**	0%
TO03 ESTs mapped to TO05 nuclear proteins		**100**	72%		**207**	70%
TO03 ESTs mapped to TO05 genome (excluding predicted proteins)		**28**	20%		**69**	23%
TO03 ESTs NOT mapped to any published TO5 sequences		**9**	6%		**18**	6%

[a]peptides of the initial LC-MS/Ms were used in searches against

[b]all proteins identified

[c]all proteins identified that map to the TO05 genome

[d]all proteins identified that map to the TO03 transcriptome

[e]original dataset in which peptides were mapped against database containing predicted proteins of TO05 genome

[f]second dataset in which peptides were mapped against a combined database of TO05 proteins and predicted proteins in TO03 transcriptome
